# *Jatropha* Diterpenes: An Updated Review Concerning Their Structural Diversity, Therapeutic Performance, and Future Pharmaceutical Applications

**DOI:** 10.3390/ph17101399

**Published:** 2024-10-19

**Authors:** Thalisson A. de Souza, Luiz H. A. Pereira, Alan F. Alves, Douglas Dourado, Jociano da S. Lins, Marcus T. Scotti, Luciana Scotti, Lucas S. Abreu, Josean F. Tavares, Marcelo S. Silva

**Affiliations:** 1 Multi-User Characterization and Analysis Laboratory, Research Institute for Drugs and Medicines (IpeFarM), Universidade Federal da Paraíba, João Pessoa 58051-900, Brazil; thalisson.amorim@ltf.ufpb.br (T.A.d.S.); luizhenriqueap@ltf.ufpb.br (L.H.A.P.); jociano.ejc2012@gmail.com (J.d.S.L.); josean@ltf.ufpb.br (J.F.T.); 2 Laboratory of Cheminformatics, Program of Post-Graduation on Natural and Synthetic Bioactive Products (PgPNSB), Health Sciences Center, Universidade Federal da Paraíba, João Pessoa 58051-900, Brazil; alvesalanx@gmail.com (A.F.A.); mtscotti@gmail.com (M.T.S.); luciana.scotti@gmail.com (L.S.); 3 Department of Immunology, Instituto Aggeu Magalhães, Fiocruz, Recife 50670-420, Brazil; ddourado.science@gmail.com; 4 Department of Organic Chemistry, Universidade Federal Fluminense, Niterói 24220-900, Brazil; abreu_lucas@id.uff.br

**Keywords:** low diterpenes, cancer, phytochemistry

## Abstract

The *Euphorbiaceae* family is a rich source of bioactive terpenoids. Among its genera, *Jatropha* is a conspicuous producer of diterpenes and includes approximately 175 species, many of which have medicinal uses. To date, 140 diterpenes from *Jatropha* (JTDs) have been reported. Given their structural diversity and notable biological activities, this work aims to highlight the pharmaceutical potential of JTDs. To achieve this goal, an extensive literature review was conducted, encompassing studies on structural elucidation through NMR and pharmacological assays, both in vitro and in vivo. Based on 132 selected papers, a thorough discussion is presented on the biosynthesis, extraction, isolation, and structural characterization of JTDs, including a compilation of their ^13^C NMR chemical shifts. The review also covers their synthetic production and biological effects. Additionally, an in silico analysis predicting the drug-likeness of 141 JTDs was carried out. Notably, the occurrence of macrocyclic diterpenes has doubled in the past decade, and the summary of their NMR data provides a useful resource for future research. Furthermore, 21 distinct pharmacological activities were identified, with potent cytotoxic effects targeting new molecular pathways being particularly significant. Recent advances highlight the contributions of modern approaches in organic synthesis and the pharmacological evaluation of natural products. The drug-likeness analysis identified JTD classes and compounds with favorable physicochemical and ADMET features for pharmaceutical development. In light of these findings, the use of nanotechnology is proposed as a future direction for continued research on JTDs, a fascinating class of natural compounds. This work opens up new avenues for the study of *Euphorbiaceae* species, particularly the *Jatropha* genus and its bioactive compounds.

## 1. Introduction

Cancer is characterized by the uncontrolled proliferation of transformed cells undergoing natural selection-driven evolution [1]. Capable of invading adjacent tissues and organs, it poses severe implications for human health and represents one of the leading causes of mortality worldwide. According to the International Agency for Research on Cancer, approximately 19,976,499 cases were reported in 2022, with lung, colorectal, liver, breast, and stomach accounting for nearly 50% of all cancer-related deaths [2]. The search for effective therapies remains a critical challenge. In this context, modern approaches have heightened interest in natural products as potential sources of novel therapeutic agents [3,4]. Given the extensive chemical diversity observed in nature, diterpenes have gained prominence due to their diverse biological activities and capacity to modulate many cellular pathways involved in tumor progression and metastasis. Some approved pharmaceuticals are derived from or inspired by diterpene structures, such as paclitaxel (Taxol) and docetaxel (Taxotere) [5,6]. The growing focus on diterpenes underscores their potential as lead compounds for anticancer drug development, emphasizing the relevance of natural products in contemporary oncology. The *Euphorbiaceae* family is a recognized source of structurally unique terpenoids, and among its 228 genera, *Jatropha* stands out for its considerable, yet underexplored, therapeutic potential.

The *Jatropha* genus includes approximately 175 species distributed across tropical and subtropical regions [7]. Many of these species have been traditionally used for medicinal purposes in Africa, Asia, and Latin America [8]. Among its secondary metabolites, diterpenes represent one of the primary classes. To date, diterpenes classified into 15 scaffold types had their occurrence reported in *Jatropha*.

Since the 1970s, diterpenes from this taxon have demonstrated activity in many biological processes, including anti-inflammatory, antimicrobial, antiviral, and, notably, cytotoxic effects against different types of cancer [9]. The antitumor potential has garnered particular interest, driving multidisciplinary efforts to enhance compound yields via organic synthesis and elucidate their mechanisms of action. Despite these efforts, only a limited number of species have had their chemical compositions investigated.

The diversity of these substances is beneficial for biological activity studies but, on the other hand, poses significant challenges for their structural elucidation [10,11]. Additionally, there is limited literature on the ADMET (Absorption, Distribution, Metabolism, Excretion, and Toxicity) parameters for these compounds, which are crucial for the discovery of innovative drugs. Moreover, although structurally diverse, many diterpenes often exhibit limited solubility. This property directly impacts biological assays and their applications in pharmaceuticals.

In light of the potential of *Jatropha* species, this paper provides readers with an updated overview focused on diterpenes, adopting a multidisciplinary approach. It encompasses their biosynthesis, structural diversity—summarizing ^13^C NMR spectroscopic data, and biological activities while exploring, for the first time, intersections with organic synthesis, medicinal chemistry, and nanotechnology. This comprehensive perspective aims to offer new insights and encourage the ongoing study of this fascinating class of natural compounds.

## 2. Literature Search and Drug-Likeness Analysis

### 2.1. Literature Search Strategy

The studies were assembled based on references focused on the isolation, structural characterization by NMR, and biological activity of diterpenes from the *Jatropha* genus (*Euphorbiaceae*). The search was conducted through SciFinder, Science Direct, Google Scholar, and PubMed. Specific search terms such as “diterpenes”, “secondary metabolites”, “chemical profile”, *Euphorbiaceae*, and “terpenoids” were employed independently and in combination with the term “*Jatropha*” and included studies ranging from 1970 to 2024 and covered 132 scientific papers.

### 2.2. Jatropha Diterpenes Drug-Likeness

The Simplified Molecular Input Line Entry System (SMILES) codes of *Jatropha* diterpenes were obtained by Jchem software [12]. Then, SMILES was converted into the Structure-Data File (SDF) format. The compiled structures were then subjected to the generation of descriptors using alvaDesc software [13]. In the current work, the druglike descriptors were considered to encode ADMET parameters for the diterpenes, and SwissADME [14] was applied as previously described [15]. As input data, the same SMILES codes used to generate molecular descriptors were used and then submitted to analysis.

The resulting data of Alvadesk and SwissADME were combined and then analyzed by principal component analysis (PCA), which was carried out using The Unscrumbler^®^ software [16]. The interpretation of the score and weight plots allowed to stablish the relation between the diterpene classes and their potential application in pharmaceuticals.

## 3. Biosynthesis

Terpenoids play crucial roles in ecological interactions, including attack, defense, and signaling in response to biotic and abiotic stresses, fulfilling vital functions in all lifeforms [17,18,19]. The isoprenoid biosynthetic pathway generates a wide range of compounds and is highly conserved [20]. This pathway follows a straightforward biogenetic structural principle, where open-chain precursors with terminal pyrophosphate groups are created, leading to the synthesis of mono-, di-, and triterpenes through intramolecular reactions. The key universal precursors, isopentenyl diphosphate (IPP) and dimethylallyl diphosphate (DMAPP), are produced by synthases, which are critical branchpoint enzymes in terpene biosynthesis [21]. In this process, DMAPP serves as an electrophile, while IPP acts as a nucleophile, allowing their combination into oligoprenyl diphosphates via prenyltransferases. From DMAPP, sequential elongation reactions with IPP first yield the monoterpene precursor geranyl diphosphate (GPP, C10), then farnesyl diphosphate (FPP, C15) as the precursor for sesquiterpenes, and geranylgeranyl diphosphate (GGPP, C20), the precursor for diterpenes (C20) [22]. 

Cyclization reactions of geranylgeranyl diphosphate (GGPP), driven by carbocation formation and the potential for Wagner–Meerwein rearrangements, result in a wide range of diterpenoid structural variants produced [23]. Based on the cyclization methods of GGPP, diterpenoids can be categorized into high or low types. In *Jatropha*, the high diterpenoids include pimarane, podocarpane, and cleistantane, characterized by their 6/6/6 ring structures formed through the classical “concertina-like: reaction. Conversely, low diterpenoids arise from “head-to-tail” cyclization, leading to a distinct group of compounds known as macrocyclic diterpenes, which are derived from casbene diterpenes [24]. 

**Figure 1:**
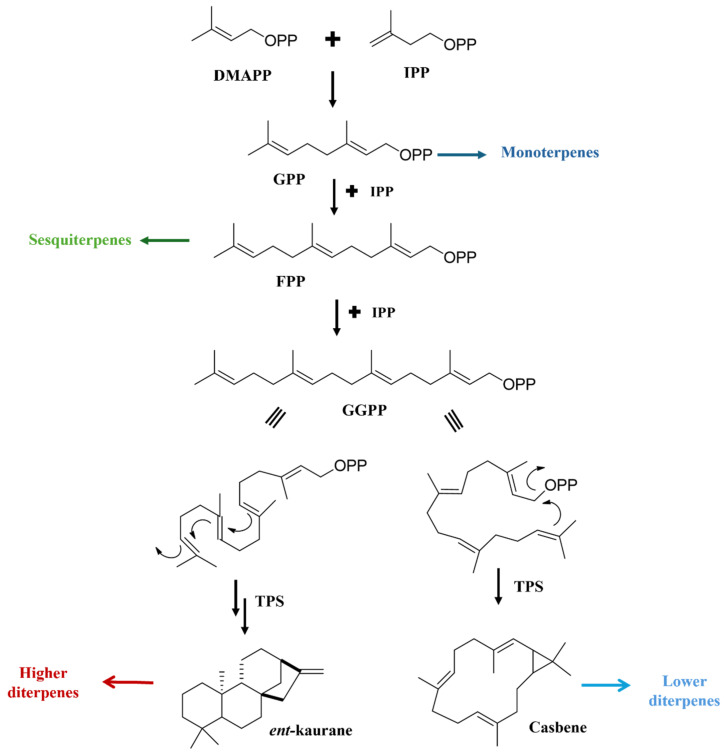
Biogenesis of *Jatropha* diterpenes from the primary precursors.

The presence of lower diterpenoids in the *Euphorbiaceae* and *Thymelaeaceae* families is a notable chemotaxonomic feature for both groups [25]. In *Jatropha*, most diterpenes are lower diterpenoids, including those with casbane, rharnnofolane, jatrophane, jatropholane, lathyrane, myrsinol tigliane, and riozolane scaffolds. In addition to the inherent chemical reactivity of this class, several factors contribute to the diversity of diterpene structures. Notably, the role of enzymes is crucial, particularly terpene synthases (TPSs) and cytochrome P450 monooxygenases (CYPs). TPSs facilitate the cyclization of the terpenes, while CYPs further modify the terpene skeleton, increasing the molecule’s polarity and introducing bioactive functional groups [26]. Figure 1 illustrates the precursors of the diterpene scaffolds identified in *Jatropha* species.

Recent studies highlighted the crucial role of terpene synthases (TPS) and cytochrome P450 monooxygenases (CYPs) in plant evolution, demonstrating their involvement in complex biosynthetic networks that deviate from traditional linear pathway models [27,28,29,30]. According to Christianson 2017 [31], substrate atoms often undergo significant changes in bonding or hybridization states during the reaction cascade, showcasing the intricate chemistry managed by TPSs. Additionally, phylogenetic analyses reveal that specific CYP subfamilies have expanded in a taxonomic manner, contributing to the diversification of particular diterpene classes. In *Euphorbiaceae*, CYP26As and CYP71Ds are notably involved in post-cyclization reactions [32,33].

Understanding these biosynthetic pathways has led to the identification of enzymes and genes related to terpenoid production in many economically and pharmaceutically significant plants [34]. In *Jatropha*, the quest for biofuel production has spurred gene identification, proteomic analysis, and other analytical studies to determine the chemical profiles and accumulation of diterpenes, particularly phorbol esters, across different genotypes and tissues of *J. curcas* [35,36,37,38]. These studies have shown higher concentrations of tiglianes in seeds, highlighting new opportunities for the metabolic engineering of *Jatropha* compounds and underscoring the chemical diversity of the species as significant producers of both high and low diterpenes. Detailed discussions on diterpene composition are presented in the following sections.

## 4. Extraction and Isolation

Extraction is essential for isolating, identifying, and separating bioactive compounds before they are analyzed [39]. The initial reports of diterpenes in *Jatropha* appeared in the 1970s. Due to their physicochemical characteristics, especially their low to moderate polarity, most diterpenes have been isolated using traditional extraction and chromatographic techniques. Although these methods can be time-consuming, they are still widely employed because of their efficiency and cost-effectiveness, even in recent efforts to discover new diterpenes. Our review found that five extraction methods are commonly used: maceration was the most frequent (62%), followed by reflux with solvents (13%), Soxhlet extraction (8%), hot solvent extraction (5%), and percolation (2%).

All these extraction methods rely on organic solvents, with n-hexane, petroleum ether, and chlorinated solvents such as dichloromethane and chloroform being commonly used. These solvents are employed both for soaking plant material and in the subsequent stages of liquid–liquid extraction and chromatographic procedures. This reliance highlights the need to explore alternative extraction methods that adhere to green chemistry principles.

Additionally, about 9% of the reviewed papers, particularly older studies, indicate that the discovery of new diterpenes was part of ongoing research. Consequently, these papers often lack detailed descriptions of the collection or extraction conditions, citing instead earlier works. Nevertheless, these references typically refer to the use of divergent methodologies, which complicates the understanding and reproducibility of the research. Therefore, there is a current need to revisit some *Jatropha* species to standardize the extraction methods for the compounds of interest.

New extraction techniques such as ultrasound and supercritical fluid offer improved yields, enhanced safety, and environmentally friendly applications, addressing classical limitations such as mass transfer and supporting the sustainable production of green chemicals [40]. The presence of phorbol esters in *Jatropha* oilseeds impaired the use of their byproducts in industry. To address this issue, modern methods have been developed for selective oil extraction. For instance, supercritical extraction has been applied to *J. curcas*, and ultrasonic-assisted extraction has been used for *J. dioica* seeds and *J. curcas*. Mechanical extraction methods for oil have also been documented [41,42,43,44]. Despite these advances, the adoption of innovative methods in discovering new chemical entities remains limited. A major challenge is the high cost of acquiring and maintaining the necessary equipment, which favors the continued use of conventional techniques.

**Figure 2:**
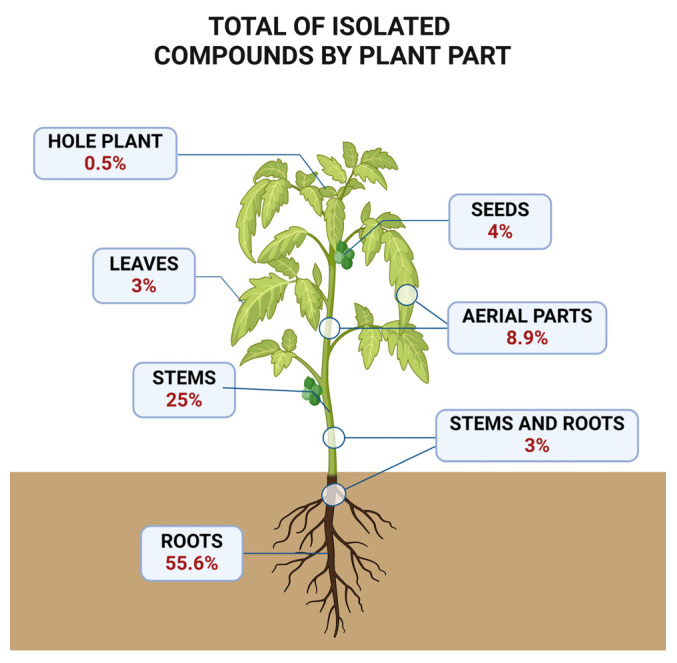
Parts of the plant identified as a source of JDT.

Higher plant roots are known for producing a wide range of secondary metabolites [45,46]. Additionally, the role of underground organs in the production of macrocyclic diterpenes is documented for many *Euphorbiaceae* species, such as *Euphorbia lathyris* [47,48]. The traditional use of *Jatropha* roots as the active ingredient in medicinal preparations has guided phytochemical studies and contributed to making them one of the main vegetal tissues chemically investigated. Figure 2 depicts the number of compounds sorted by plant parts. 

In *Jatropha*, the accumulation of phorbol esters in specific tissues in response to various stimuli has been documented for *J. curcas* [49]. According to de Almeida et al. (2021), the products of the casbane synthase (CS) gene, a terpene synthase (TPS) enzyme responsible for the initial step in the biosynthesis of macrocyclic diterpenes, are found exclusively in the roots of this species [50]. Additionally, the traditional use of roots in folk medicine has historically driven phytochemical research [51]. These observations may help explain the extensive variety of diterpenes isolated from the underground parts of *Jatropha* species

## 5. Chemical Diversify and Structural Characterization of *Jatropha* Diterpenes

Once isolated, determining the chemical structure is essential for discovering new bioactive molecules. Advances in spectroscopic and spectrometric techniques have greatly enhanced the ability to identify these compounds. Among these techniques, Nuclear Magnetic Resonance (NMR) stands out as a crucial method for elucidating natural compounds. Improvements in both instrumentation and experimental procedures have reduced analysis times and increased sensitivity, allowing for a more efficient examination of materials in microgram quantities. The integration of NMR techniques with computational methods has facilitated the rapid chemical profiling of extracts, expanding their application in metabolomics. This approach supports the identification of novel active compounds, assessment of activity–metabolite correlations, and ensures the quality control of medicinal plants. An additional advantage of the modern approaches, combining NMR data and theorical analysis the determination of stereochemistry in complex molecules have been improved, leading current studies to increase their focus on the structural revision of natural compounds [52,53,54,55,56].

Studies that compile NMR chemical shifts, particularly ^13^C, of compounds isolated from specific plant groups or chemical classes have greatly benefited the natural product chemistry community. These studies provide crucial information on molecular patterns that assist in the structural elucidation of complex molecules, such as diterpenes, which can be challenging. While the ease of accessing references and the availability of software tools for predicting NMR spectra have made such studies more meager, databases of experimental NMR results remain highly valuable [57]. They support various applications across different research fields, including enhancing prediction algorithms and facilitating dereplication, thus accelerating the discovery of new bioactive molecules [58].

To date, 140 diterpenes have been described in the genus *Jatropha*, doubling over the past decade [59]. These include both higher and lower terpenoids, found across approximately 170 botanical occurrences. The higher terpenoids encompass cleisthantanes, pimaranes, and podocarpones. Macrocyclic diterpenes constitute the majority of the compounds in this genus, contributing significantly to the extensive chemical diversity observed. Our survey identified lower diterpenes from 12 different scaffolds, including seco compounds and other derivatives.

**Figure 3:**
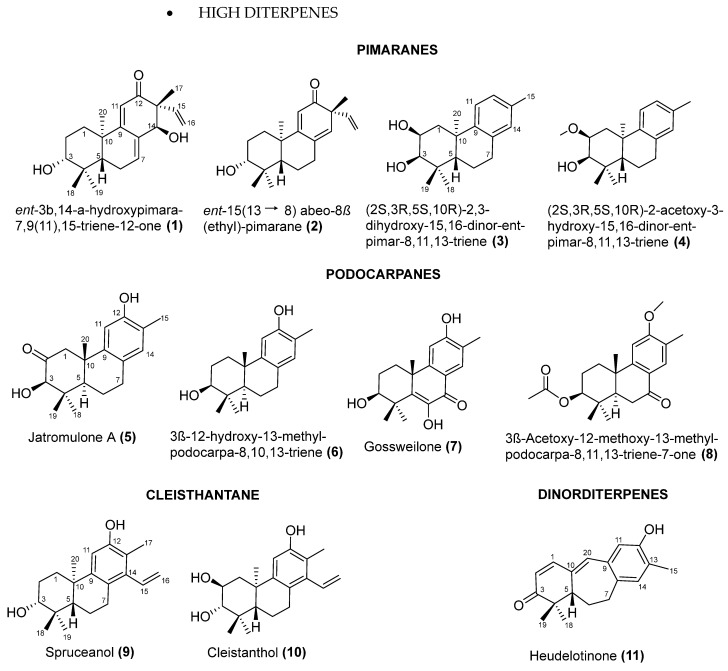

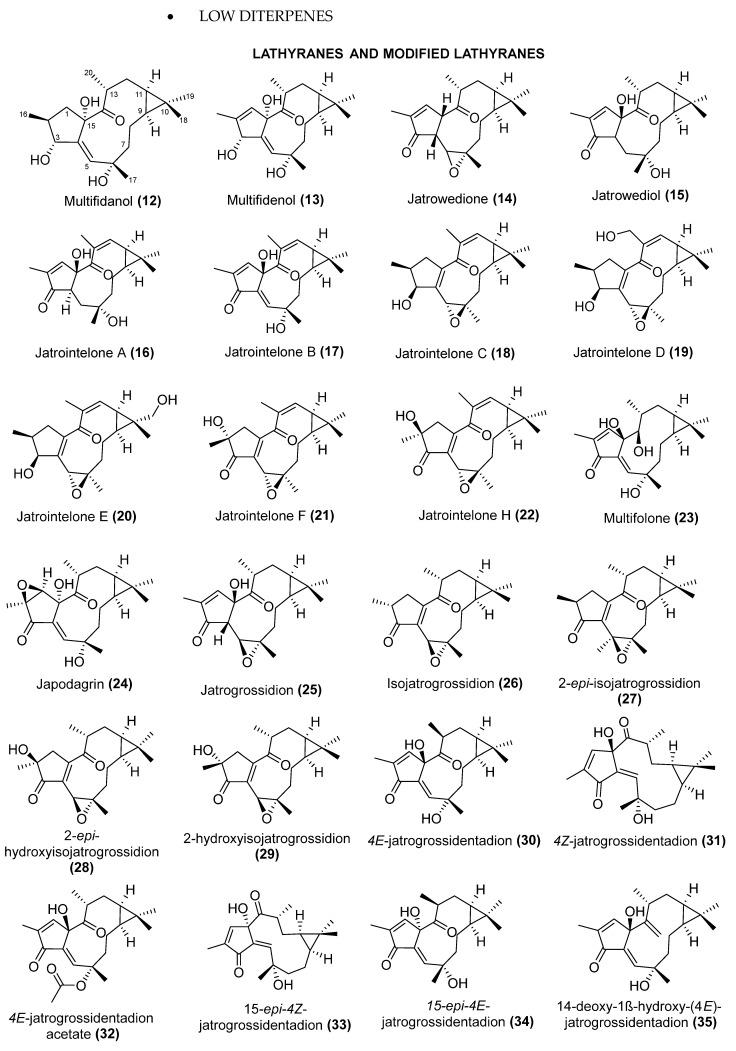

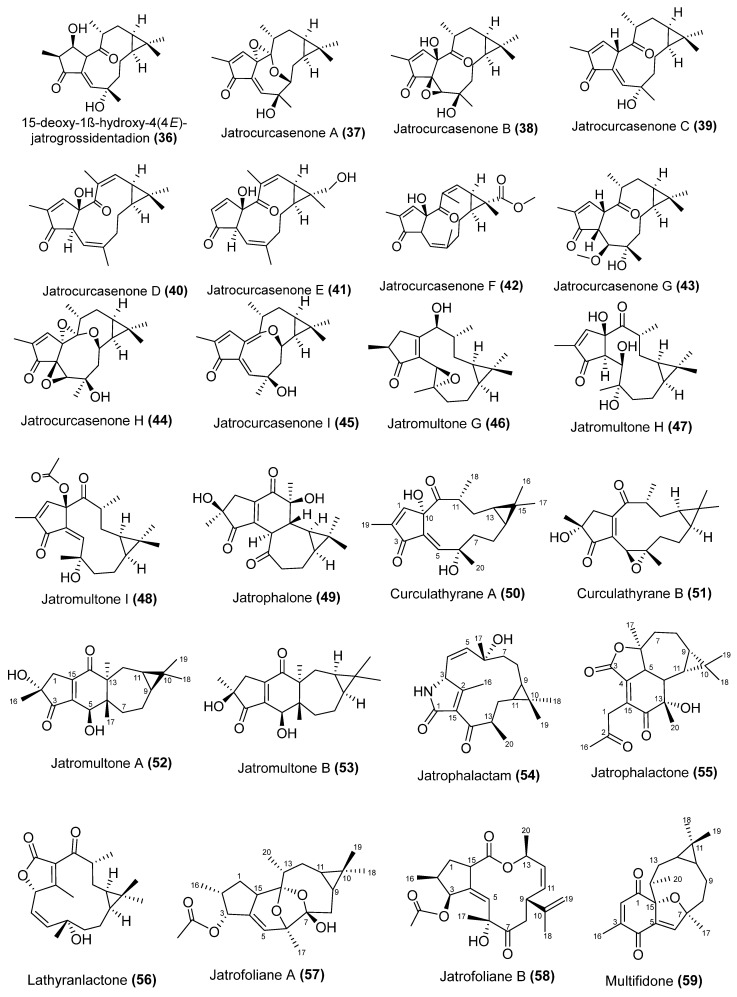

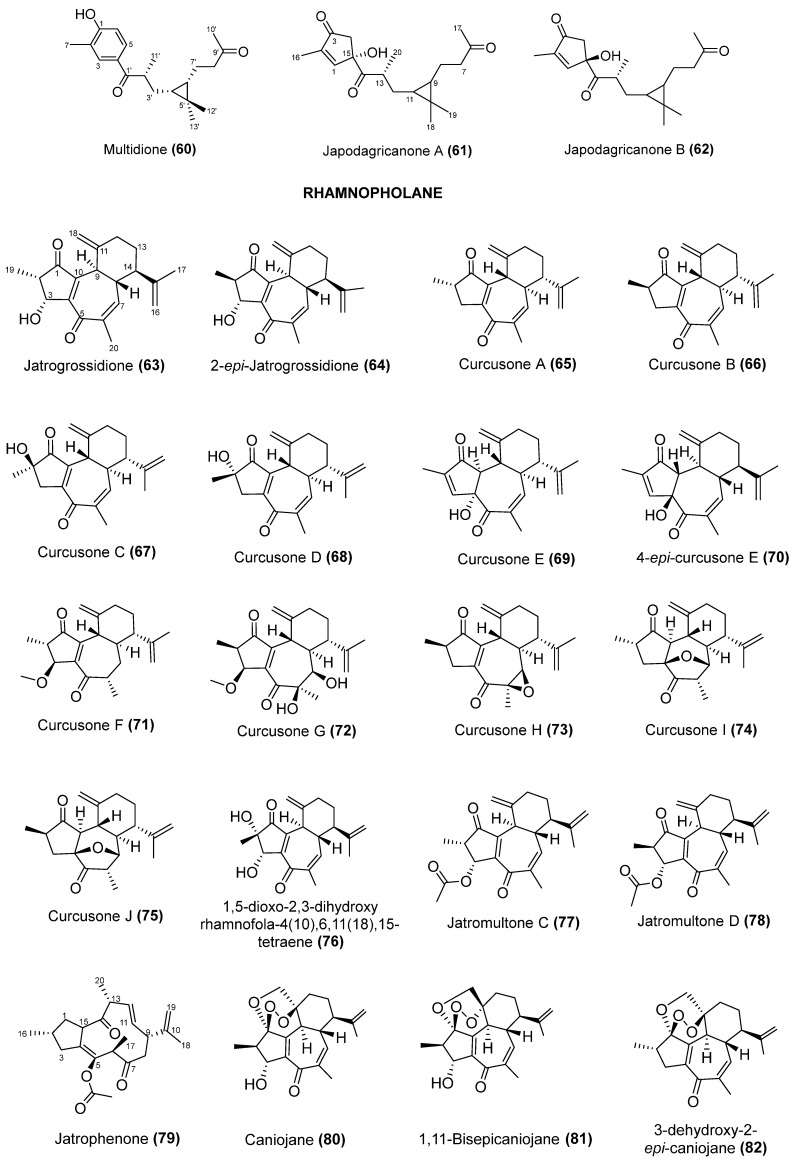

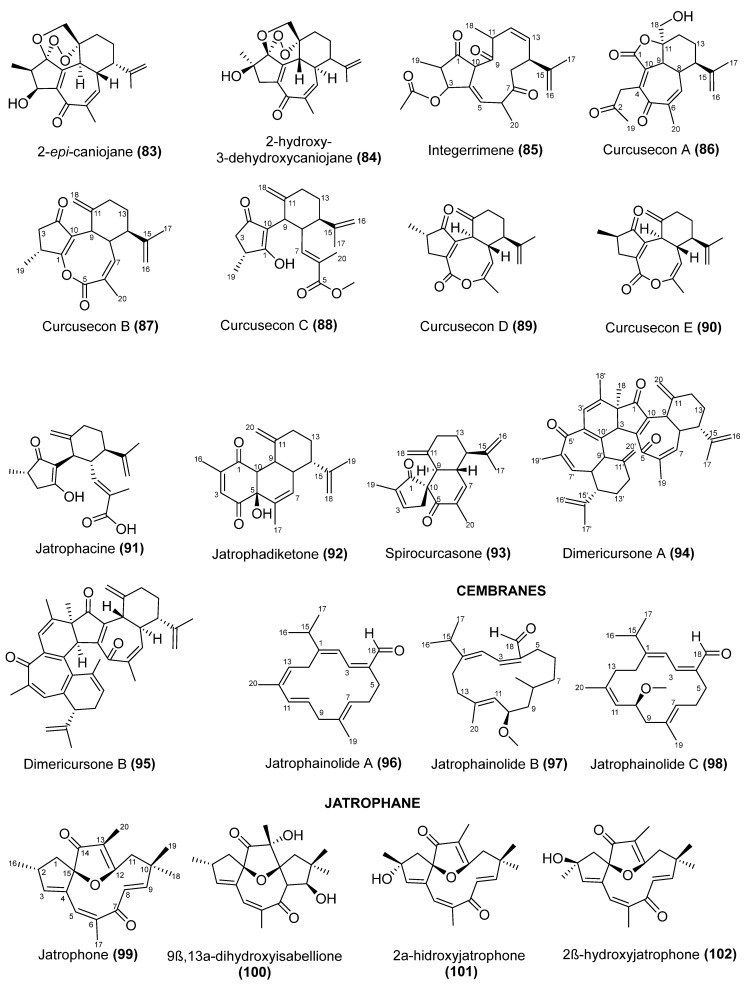

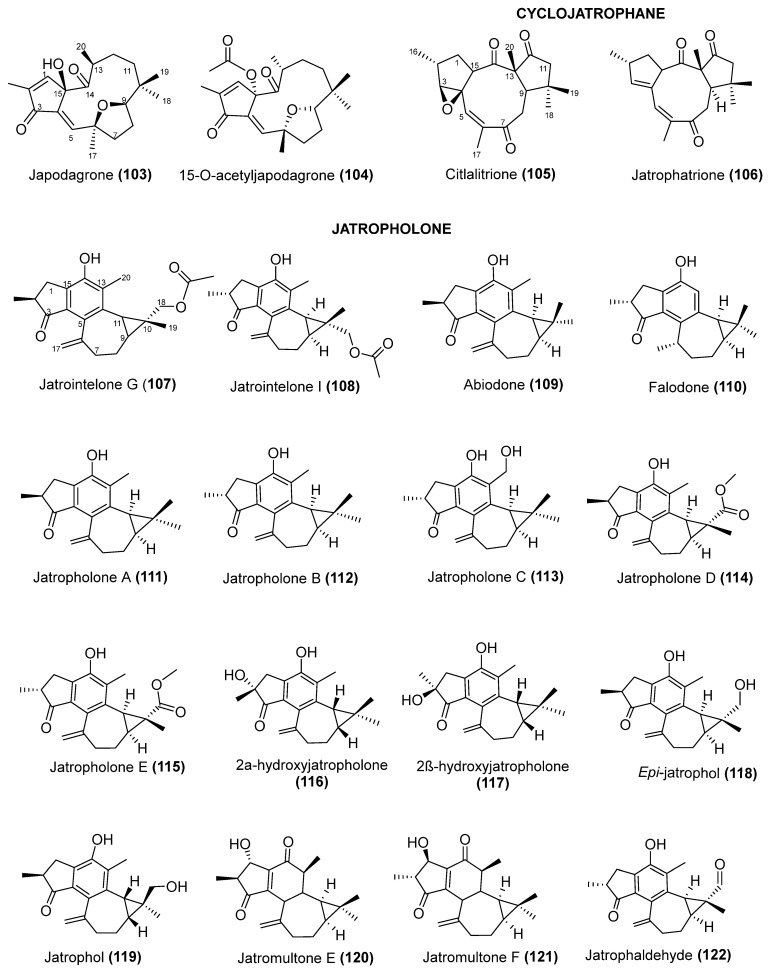

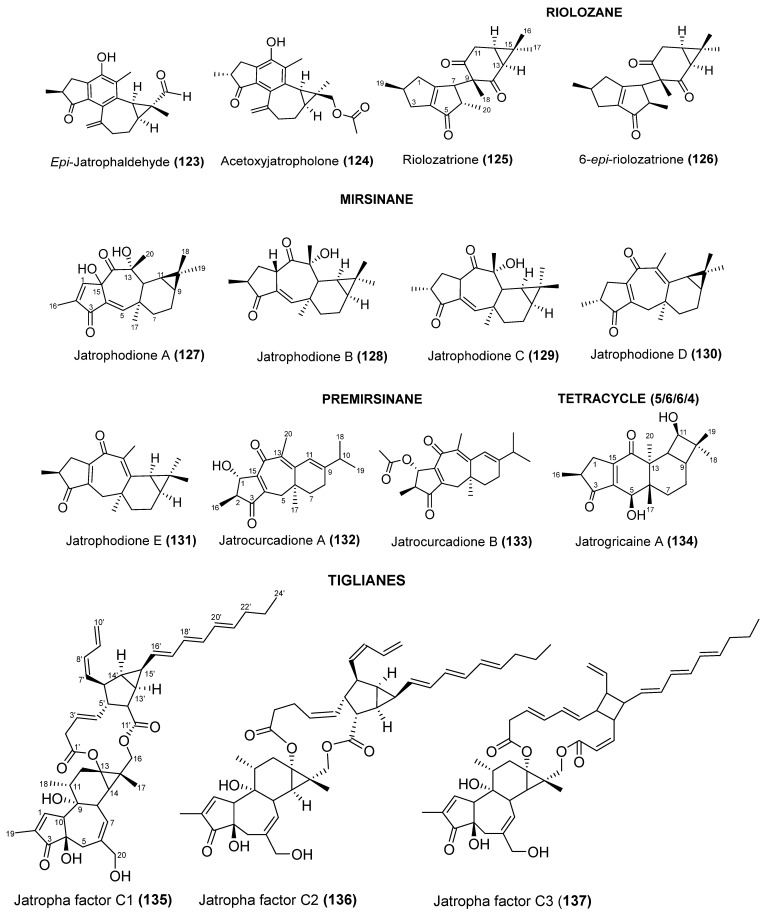

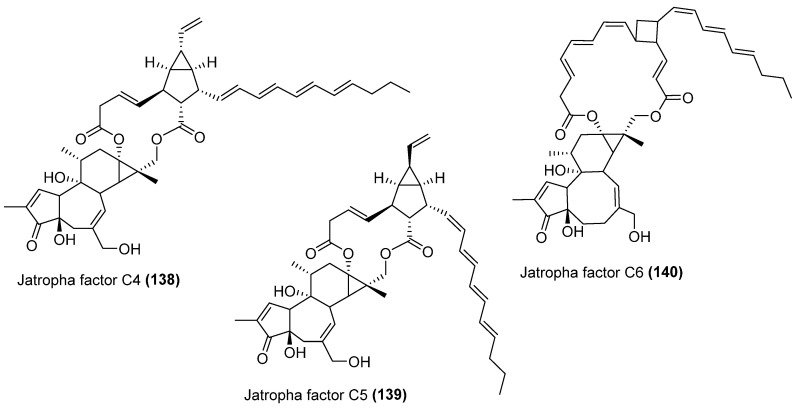
The 140 structurally diverse diterpenes isolated from *Jatropha* species.

The structural elucidation and identification of these isolated compounds typically require a combination of spectroscopic techniques, including 1D and 2D NMR, mass spectrometry (MS), and infrared (IR) spectroscopy. Due to practical constraints, it is not feasible to present and discuss the NMR data for all structural variations of *Jatropha* diterpenes isolated thus far. Therefore, this review compiles the structures, as illustrated in Figure 3, and provides their corresponding ^13^C NMR chemical shifts, as detailed in Table 1. Additionally, all data will be also available in a free open natural products database, SistematX (http://sistematx.ufpb.br), offering a foundation for future research on *Euphorbiaceae* diterpenes. The SistematX Web Portal, introduced in 2018, documents crucial information about plant secondary metabolites and includes 9514 unique compounds arising from 20,934 botanical occurrences [60].

## 6. Chemical Synthesis of *Jatropha* Diterpenes

The unique structural features of natural products, coupled with their diverse biological activities, have demanded the development of new strategies in organic synthesis [110]. In this context, terpene synthesis is a leading area in modern synthetic chemistry. For *Jatropha* diterpenes, while lathyranes are the most frequently isolated compounds, the total synthesis of jatrophanes and rhamnofolanes has been more thoroughly documented. Compounds such as jatrophone, jatrophatrione, citlalitrione, jatropholanes A–B, and, more recently, spruceanol and curcusone series have reported synthetic routes

To highlight the impact of organic synthesis in this field, we traced the developments starting from the 1970s, particularly focusing on the total synthesis of jatrophone. Initially isolated from *J. gossypiifolia* L., jatrophone was the first compound of its class to be synthesized. Its macrocyclic structure and 3(*2H*)-furanone ring, also found in other cytotoxic compounds of that era, prompted further activity studies and would lead to the identification of new antitumor agents. These early efforts sparked numerous attempts, many of which were unsuccessful, as detailed by Smith III 1984 [111]. The same structural features that intrigued researchers also presented significant challenges until jatrophone was eventually synthesized, as briefly discussed below.

The first total synthesis of jatrophone was accomplished through a three-phase project. The initial phase focused on developing the 3(*2H*)-furanone ring, the second on constructing the macrocycle, and the third on controlling the reaction’s stereochemistry to closely mimic the natural compound. This project successfully synthesized jatrophone and several related compounds, including normethyljatrophone, its cis and trans derivatives, and hydroxylated derivatives at C2 (2α and 2β-hydroxyjatrophone) [112,113]. In the 1990s, new protocols for synthesizing jatrophone and its epimers were introduced. The first protocol involved palladium-catalyzed carbonylative coupling of vinyl triflates with vinyl stanols to form the macrocycle, while the second used a Wadsworth–Horner–Emmons variant for constructing the C ring of jatrophone, employing a Pd-catalyzed cross-coupling to incorporate the C5-C6 double bond with the necessary Z stereochemistry and forming the macrocycle through condensation of an acetylenic aldehyde [114,115].

Since then, the scope of jatrophane diterpene synthesis has expanded beyond a single compound. Synthetic chemists have shown interest in other bioactive substances, particularly those from the *Euphorbia* genus, such as those found in *E. characias*. A comprehensive discussion of the strategies and reactions used up to the last decade can be found in previous publications [116,117,118]. Following jatrophone, the synthesis of jatropholanes A–B, also isolated from *J. gossypiifolia* L., was achieved. This synthesis involved a 12-step process with a 6% overall yield starting from 1-pyrrolidinocyclopentene and demonstrated the application of high-pressure Diels–Alder reactions in natural product synthesis [119].

After a hiatus, the synthesis of *Jatropha* diterpenes was revitalized with the production of cyclojatrophanes, jatrophatrione, and citlalitrione, as reported in 2003. The authors developed a linear synthesis strategy involving a 20-step sequence to achieve these compounds. Through retrosynthetic analysis, they identified key factors related to forming the tricyclic backbone and subsequently introduced the remaining functional groups. The protocol began with an anionic oxy-Cope rearrangement, which generated a tetracyclic structure. This was followed by hydroxylation at C12 and C14 through intramolecular hydrosilylation. The resulting allyl alcohol from Treibs enabled regioselective dehydration, and the 1,3-diketone segment was then added to produce jatrophatrione. The subsequent peracid oxidation of jatrophatrione yielded (±)-citlalitrione [101].

The recent literature has introduced protocols for the synthesis of spruceanol, a cleistantane diterpene, and curcusones A–D, J, and I, macrocyclic diterpenes of the rhamnofolane type. For spruceanol, the synthesis was achieved in 13 steps, starting from the naturally occurring *ent*-labdane diterpene andrographolide, with an overall yield of 9%.

The synthesis involved Lewis acid-controlled regioselective Diels–Alder cycloaddition and Wittig olefination techniques to construct the skeleton of the target compound [120]. 

Regarding curcusones A–D, three different approaches to their synthesis have been reported. The first method involves constructing the 5-7-6 carbocycle core of the curcusones using complementary Stetter annulation or ring-closing metathesis disconnections, with a key aspect being the use of a cross-electrophile coupling strategy. An alternative approach from the same period employs Suzuki coupling, intramolecular cyclopropanation, and a crucial divinylcyclopropane rearrangement for the synthesis of curcusones A–D. The third approach, described in another paper, utilizes a thermal [3,3]–sigmatropic rearrangement and a novel cascade reaction catalyzed by FeCl_3_ to rapidly build the essential cycloheptadienone core of the curcusones, resulting in curcusones A and B in 9 steps, C and D in 10 steps, and dimericursone A in 12 steps. Additionally, curcusones I and J were synthesized using a tandem gold-catalyzed furan formation, furan-alkene [4+3] cycloaddition, to create the fused 5,7-ring system, and an exo-Diels–Alder reaction to form the 6-membered ring [86,121,122,123].

The synthesis of biosynthetic precursors like casbane, as well as diterpenes with different backbones such as tiglians and myrsinans, highlights the significance of *Euphorbiaceae* terpenoids in the domain of organic synthesis [124,125,126]. In this regard, the field’s contributions to natural product research have been solidified through efforts to achieve higher yields of these compounds, expand their structural diversity, and improving the comprehension of their biosynthesis. Examples of this progress include bioinspired synthesis, chemoenzymatic methods, and synthetic biology approaches for terpene production [127,128,129]. Furthermore, the successful synthesis supports ongoing biological studies, as will be discussed in the following section.

## 7. Biological Activity

The review of the biological analyses of the isolated diterpenes identified 21 different activity models and revealed that only 15 compounds within this group remain untested. This situation contrasts with other natural product classes, such as pregnane glycosides from the *Apocynaceae* family, which, despite their extensive chemical diversity, have been minimally explored pharmacologically [130]. This discrepancy might be attributed to the availability of these compounds through organic synthesis and their occurrence in other *Euphorbiaceae* species, particularly *Euphorbia* and *Croton*, which are more extensively studied both chemically and biologically [24,131,132].

Among the many assays conducted, cytotoxicity against tumor cell lines (in vitro) is the most commonly tested, with lathyranes and rhamnofolanes being the most prominent diterpene groups, followed by jatrophane. A summary of the most potent compounds is provided in Table 2. Although promising, well-defined structure–activity relationship models for these diterpene classes are still scarce in the literature. Factors such as the heterogeneity of cell lines and low yields may contribute to the limited progress on this issue.

Based on the aforementioned, three JTD scaffolds (rhamopholanes, jatrophane, and lathyranes) stands out as the most cytotoxic compounds identified in the genus. Their activity also includes effectiveness over some prevalent types of cancer with the highest mortality rates, such as breast, lung, and colorectal carcinoma [2]. Their effectiveness was assessed mainly by colorimetric assays. Among the top ten compounds (*vide supra*), MTT was the most applied. The advantages of using colorimetric assays are attributed to their speed, cost, and sensibility, offering a quick way to quantify cell viability, proliferation, or death [137]. Furthermore, in vitro cultures provide biological relevance and precise experimental control, allowing to test their selectivity and efficacy in many types of cancer and enabling more detailed investigations of the mechanisms of action [138]. The combination of both approaches is useful in discovering new compounds with antitumor potential, starting with fast screenings through colorimetric assays over cell lineages and advancing to in vivo models.

Isolated from the roots of *J. curcas*, the rhamnofolanes curcusones A–D were first discovered in 1986, though the interest in these compounds has only recently been renewed [86]. Research on these compounds offers significant structural insights into their cytotoxic activity. Notably, the formation of the seven-membered ring through the C8–C14 connection and the presence of the dienone system in ring B are crucial. The relative configuration of Me-19 and the hydroxyl group at C-2 influence their potency against hepatocellular carcinoma and lymphoma cell lines, although their activity is maintained. For lathyranes, jatrogrossidion derivatives, the presence of the 5/11/3-ring system, the epoxy, or the double bond at C-5 were observed as important elements for maintaining activity, since the presence of 5-OH was harmful [77,88].

In terms of mechanisms of action, chemical proteomics has emerged as a cutting-edge method for systematically screening and identifying the protein targets of active small molecules [139]. This approach integrates natural product chemistry and probe design by synthesis with proteomics and has been effectively applied to curcusones. Specifically, curcusone C has been shown to induce mitochondrial damage and activate the mitochondrial apoptosis pathway in prostate and endometrial cancer cells. Its mechanism involves the TGF/Smad signaling pathway and disrupts the Bax/Bcl-2 balance by inhibiting the expression of the target protein, poly(rC)-binding protein 2 (PCBP2). Additional studies suggest that curcusone C may interact with Telomeric Repeat Factor 2 (TRF2), inducing a telomeric DNA damage response, inhibiting tumor cell proliferation, and causing cell cycle arrest, which leads to tumor cell apoptosis [140,141,142,143]. The ability to target specific proteins represents significant progress in cancer therapeutic strategies.

Curcusone D has been identified as an inhibitor of the BRCA1-associated ATM activator 1 (BRAT1), which is a significant and previously “undruggable” target. This inhibition results in an impaired DNA damage response and reduced cancer cell migration while also enhancing the efficacy of the DNA-damaging drug etoposide. Additionally, in multiple myeloma, curcusone D induces cell growth inhibition and apoptosis by acting as an inhibitor of the ubiquitin–proteasome pathway. The identification of curcusone D targets highlights the potential of natural products to pave new avenues for cancer treatment and overcome resistance to conventional chemotherapy agents [86,144]. Similarly to curcusone D, jatrogrossidione also displays antitumor activity mediated by cell growth inhibition and apoptosis. According to Zhang et al. 2018 [136], jatrogrossidione inhibits the proliferation of RKO cells via the induction of G2/M phase arrest and causes a remarkable increase in cellular apoptosis in a dose-dependent manner. However, differing from curcusone D, the molecular targets involved in that activity remain undescribed.

For jatrophane-type diterpenes, jatrophone has been shown to inhibit the proliferation of MCF-7/ADR cells at low micromolar concentrations through a mechanism involving the PI3K/Akt/NF-κB pathway [135,145]. The activation of PI3K/AKT is known to promote tumor cell growth, proliferation, invasion, and metastasis while inhibiting apoptosis. In contrast, although lathyranes, such as jatrogrossidion derivatives, display significant cytotoxic potential, the precise mechanisms underlying their activity remain unclear, but some authors associate that as a response to P-glycoprotein inhibition [146].

**Figure 4:**
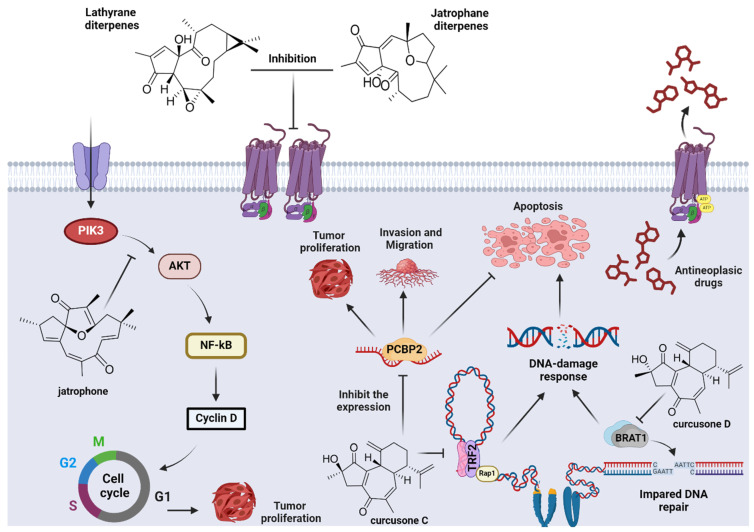
Main targets involved in antitumoral and MDR activities of JDT.

Lathyranes and jatrophanes have demonstrated efficacy as anti-MDR (P-glycoprotein-mediated multidrug-resistant) agents. Research involving *Euphorbiaceae* species has been instrumental in identifying the pharmacophoric elements for these scaffolds and developing QSAR models for these classes [147,148,149]. Despite their potential, only a limited number of JTD have been assessed for their effectiveness against MDR. Targeting this molecular mechanism is therapeutically significant, because it addresses resistance to various chemotherapeutic agents and enhances cytotoxic effects by both increasing the intracellular concentration of antineoplastic drugs and restoring immunogenic cell death [150,151]. Figure 4 provides an overview of the primary targets.

Beyond their cytotoxic properties, some diterpenes have demonstrated effectiveness against diseases such as leishmaniasis, tuberculosis, and malaria. Structural modifications of jatrophone have shown that the olefinic bond between C8 and C9, along with the overall scaffold, contributes to its antimalarial activity. Compounds like caniojane and other peroxide-bridged diterpenes have exhibited strong anti-plasmodial effects. Additional research supports the potential of diterpenes in combating various neglected tropical diseases [152,153], although studies specifically focusing on macrocyclic diterpenes are limited compared to other classes like sesquiterpene lactones found in *Asteraceae* or synthetic compounds [154,155,156].

The numerous in vitro studies highlight the benefits of these methods in natural product research. However, there is a notable lack of in vivo, ex vivo, or alternative model studies. Some compounds, such as 9β,13α-dihydroxyisabellione, jatrophone, and jatropholones A–B, have been evaluated for their gastroprotective effects. For instance, jatropholone B and jatrophone demonstrated dose-dependent activity, reducing lesions by 91% and 93% at the highest concentration (100 mg/kg), respectively. Additionally, the effects of jatrophone on smooth and cardiac muscle contraction have been reported, showing that it directly inhibits muscle contractions, with its potency varying depending on the tissue and stimulus used [157,158,159]. 

Compounds such as jatrophone, jatropholones A–B, and spruceanol have been extensively tested for various activities and have yielded promising results. Although these findings raise questions about selectivity, they also reveal structural patterns with potential applications to different molecular targets. This has led to the development of the concept of multitarget drugs, which are compounds capable of interacting with multiple targets simultaneously. Such an approach holds promise for addressing complex diseases, offering advantages such as increased efficacy, improved safety profiles, and simplified administration [160,161]. In this context, JTD could be considered promising candidates. Understanding their mechanisms of action and assessing their toxicity are crucial for exploring their potential in pharmaceutical applications.

The insights presented in this review reinforce the notion that the search for new natural bioactive substances should continue alongside the study of well-established molecules. A comprehensive list of reported activities for each compound is available in the Supplementary Materials (Table S1 Supplementary Materials). For further details on the testing and biological activities of extracts and fractions, readers are encouraged to consult previously published papers [9,51,162,163].

## 8. Drug-Likeness Properties

It is well established, in one guise or another, that natural products have historically been essential in drug discovery, consistently providing new therapeutic agents. The biological pressures exerted by nature contribute to molecular diversity and refine these compounds to interact with molecular targets. For example, more than 30% of all FDA-approved new molecular entities are derived from or inspired by microbial and plant species [164,165]. However, advancing a molecule through clinical trials also depends on its physicochemical characteristics, collectively known as its drug-like profile.

The concept of “drug-like” was developed based on the physicochemical properties of approved drugs, such as molecular weight, hydrophobicity, and polarity. Classic studies identified common features among these drugs and proposed ranges for these properties, one of the most well-known being the Rule of Five (Ro5) [166]. Substances that fall within this range are considered “drug-like” and are likely to exhibit favorable bioavailability. Assessing drug-like attributes has become a standard practice in medicinal chemistry. The development of advanced algorithms to predict these properties has been applied to large molecular datasets, including natural product libraries (NPLs), to aid in the discovery and selection of promising candidates [167,168,169].

Notable among the methodologies used to construct NPLs are knowledge-driven approaches that integrate chemical and taxonomic information. These methods help in pinpointing regions within chemical space where bioactive compounds are likely to cluster. Additionally, understanding the relationship between biosynthetic gene clusters and the molecular structures of secondary metabolites can significantly influence natural product discovery and engineering [170,171].

**Figure 5:**
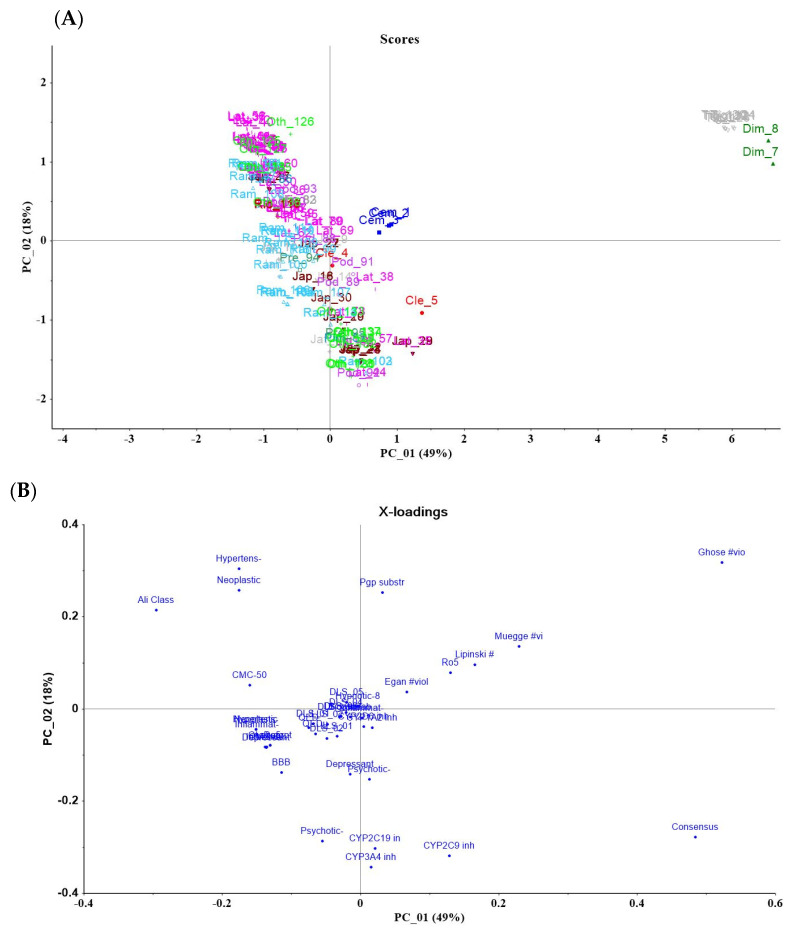
PCA score plots of JTDs (**A**). The compounds were sorted by the scaffold indicated by colors and labels containing the initial letters of each group followed by the number on the ID list. Weight plots (**B**) demonstrate the qualitative ADMET descriptors and chemical space comprised by CMC (80% or 50%) or one of the seven drug class subsets.

Literature reviews are valuable resources for knowledge-driven approaches. A recent systematic review by Obende et al. (2024) on secondary metabolites from *Croton* (*Euphorbiaceae*) included predictions of their ADME/Tox properties [172]. The study revealed that 38% of *Croton* compounds adhere to the Rule of Five (R05), and 76.37% exhibited physicochemical profiles similar to those of FDA-approved drugs, clustering within the same chemical space. Building on the advantages of NPLs and drug-like properties for identifying potential hits, we have assembled a small dataset of JTD and performed in silico predictions of their ADMET and drug-likeness. The results are illustrated in a PCA (principal component analysis) plot shown in Figure 5.

The analysis of the score graph in Figure 5A reveals a total variance of 67%, clearly distinguishing cembrane-type diterpenes, tiglian, and rhamnopholan dimers from the other compounds. When these findings are compared to the loading plot graph (B), it becomes evident that these substances fall outside the optimal range for drug-like compounds, as predicted by various algorithms. These include the Rule of Five (R05), Egan’s rule, Ghose’s rule, Veber’s rule, and Muegge’s rule, alongside the noted violations (represented by #). The output parameters consider factors such as the octanol/water partition coefficient (log Po/w), molecular weight (MW), the number of hydrogen bonds donated/accepted by the solute in an aqueous solution, the number of rotatable bonds (RB), and the predicted brain–blood partition coefficient (BBB) [14]. 

The literature identifies Protein Kinase C (PKC) as a molecular target for tiglian diterpenes. While advancements in the structural characterization of PKC isoforms and variations in their expression in tumor cells suggest that tiglians may exhibit selective cytotoxic activity, prior studies on enriched fractions indicate that the tiglian compounds present in *Jatropha* oil seed possess irritant properties and inherent toxicity. As a result, these compounds are generally excluded from consideration as promising candidates for pharmaceutical development [173,174]. 

Another variable that plays a significant role in the differences observed in the PCA is “consensus”, which reflects the average LogP (octanol–water partition coefficient) predicted by the previous mentioned algorithms. In summary, compounds with higher LogP values tend to cluster in quadrants 2 and 4, while dimers and tiglians display extreme LogP values, respectively. Although more advanced methods for calculating a substance’s lipophilicity are available, LogP remains a standard parameter correlated with bioavailability. This analysis demonstrates that, compared to drug databases focused on specific therapeutic activities, the compounds with the most favorable profiles are grouped within quadrants 1 and 3.

A careful examination of the molecular descriptors suggests that the variables located in the first quadrant (loading plot B) fall within the property range between 80% or 50% of the total CMC (chemical space of its subsets), which includes approved drugs such as; antineoplastic (Neoplastic-50), antihypertensive drugs (Hypertens-50), and Ali. As the distribution moves toward the center of the graph (score plot A), the threshold increases, along with the lipophilicity, reaching at least 80% similarity to the same classes, including anti-inflammatory and anti-infective drugs (Neoplastic-80, Hypertens-80, Antiinflammat-80, and CMC-80). Consequently, as represented by the ADMET variables (Bioavailability, GI Absorption, and R05), this set complies with Lipinski’s Rule of Five (cR05), showing the optimal values for GI absorption and good estimated bioavailability. Among the promising substances are the rhamnopholanes, such as curcusones C–H, caniojane, and its derivatives, followed by jatrophanes derived from jatrophone, multifidone, and *epi-*jatrophol—a lathyrane and jatropholone, respectively 

The alignment of ADMET predictions with the descriptors from drug banks also suggests that molecules capable of crossing the blood–brain barrier (BBB) would be candidates for the development of antidepressant and antipsychotic agents, as indicated by the labels on the loading plot (BBB, Depressant-50, and Psychotic-50). The structural features of the diterpenes may account for some of these findings, such as the arrangement of the rings, which resembles known antidepressant compounds. However, it is important to note that, as the distribution of the molecules shifts toward the fourth quadrant, their inhibitory potential for CYP enzymes—specifically, CYP3A4, CYP2C9, and CYP2C19—also increases. These enzymes play a critical role in the metabolism of drugs and xenobiotics and are implicated in numerous clinically significant drug interactions. Expanded versions of Figure 5A,B are provided as supplementary information for further analysis.

## 9. Perspectives

The macrocyclic scaffolds represent the primary structure of diterpenes found in *Jatropha*. Despite continuous research efforts, their discovery still largely depends on traditional extraction and isolation techniques. However, advances in dereplication methods using MS and NMR have significantly expanded the potential for accelerating and prioritizing the discovery of novel compounds, particularly among *Euphorbiaceae* diterpenes. 

When evaluating their bioactive potential, as previously mentioned, most studies have focused primarily on in vitro assays, especially for measuring cytotoxicity against cancer cells. One notable limitation is that many compounds have not been tested for cellular viability in normal cells. Furthermore, preclinical and clinical studies are notably absent, yet these steps are crucial for the development of phytomedicines and pharmaceuticals. Issues such as low aqueous solubility, limited bioavailability, potential non-selective toxicity, and pharmacokinetics also pose challenges. Given these limitations, nanotechnology could offer promising strategies to overcome these obstacles and enhance the therapeutic potential of JTD.

Nanotechnological systems, ranging from 1 to 1000 nm, offer the potential to enhance the biocompatibility properties of these molecules, shielding them from biological and external environmental factors and increasing their permeability across physiological barriers, thereby boosting bioavailability. By promoting a controlled release profile, these systems can also reduce toxicity by increasing the selectivity toward tumor cells.

In this review, several cytotoxic compounds have shown significant potential, particularly from the rhamnofolan, jatrophane, and lathyrane classes. Among them, curcusone C and D stand out as the most potent. While in silico predictions suggest they exhibit favorable aqueous solubility, other limitations, such as those previously mentioned, must still be addressed. For these compounds, polymeric nanoparticles created through a double emulsification process offer a promising solution. These systems consist of an internal aqueous phase (W1), where the molecule is dispersed in an organic phase (O) containing a solvent that dissolves the polymer. This forms a primary emulsion (W1/O), which is then dispersed in a secondary aqueous phase (W2), resulting in a multiple emulsion (W1/O/W2) through a process of emulsification and solvent evaporation. This approach could lead to a high encapsulation efficiency of these diterpenes, enhancing their therapeutic potential.

For diterpenoids with moderate aqueous solubility, such as jatrophone, jatromultone D, and jatrogrossidione, lipid-based delivery systems like oil-in-water (O/W) microemulsions (10–100 nm) and nanoemulsions (50–500 nm) are recommended. These systems consist of oil droplets dispersed in an aqueous phase stabilized by surfactants. They differ in the surfactant concentration and the thermodynamic nature of the systems. This configuration not only enhances the solubility of the encapsulated molecule but also increases the surface area, thereby boosting biological activity.

For more hydrophobic molecules, lipid nanoparticles, such as solid lipid nanoparticles (SLNs) and nanostructured lipid carriers (NLCs), are viable alternatives. These systems can encapsulate diterpenes within a lipid matrix composed of either solid lipids (in SLNs) or a mix of solid and liquid lipids (in NLCs). Additionally, core–shell nanoparticulate systems, such as nanocapsules, offer another promising approach. These have an oily core that solubilizes the molecule and a polymeric coating that provides protection and controls release. Beyond encapsulation, these systems allow for the surface adsorption of diterpenoids, as well as functionalization with substances that promote stealth properties, targeted delivery to specific cells, and enhanced cellular uptake.

Thus, considering the diverse range of nanotechnological systems available, their application to these compounds is strongly encouraged as a new and promising strategy for harnessing their biological properties more effectively. This approach holds the potential to advance towards a more precise and translational use, paving the way for the development of phytomedicines that can be efficiently produced and utilized.

## 10. Conclusions

The current review highlights the extensive chemodiversity of JTD, showcasing their biosynthetic pathways, extraction and isolation, structural features, and biological effects. A total of 140 JTDs were considered for this work, as the compilation of their ^13^C NMR chemical shifts provided essential information for guiding the discovery of new chemical entities from *Jatropha* species. The pharmacological importance of JTDs is emphasized by their ability to interact with novel molecular targets and exhibit potent bioactivities, which strengths their role in the development of potential therapeutic agents, especially against cancer. In addition, the role of organic synthesis for the obtention of JTD illustrates modern strategies that have led to some of the most promising compounds reported in the genus, which includes jatrophone and curcusones A–D. Allied to in silico analysis, the current work provides valuable insights into the ADMET properties and drug-likeness of JTD, offering a foundation for future pharmaceutical applications, particularly as new cytotoxic agents. Among the classes covered, the rhamnopholanes stands out as one of the most interesting scaffolds to further development due to their favorable physicochemical properties when compared to approved drugs and cytotoxic potential. In addition, the authors envision the use of current technologies to guide the discovery of new diterpenes from natural sources and nanotechnology approaches to improve the biopharmaceutical properties of the most active molecules. These results underscore the need for continued research in this area and pave the way for future research on *Euphorbiaceae* species, mainly those within the *Jatropha* genus.

## Figures and Tables

**Table 1:** ^13^C chemical shifts of JTD, ẟ in ppm.

**Compound**	**1**	**2**	**3**	**4**	**5**	**6**	**7**	**8**	**9**	**10**	**11**	**12**	**13**	**14**
**C1**	34.4	36.0	40.4	36.5	51.7	37.0	34.5	35.9	37.3	44.4	149.6	42.0	128.6	156.0
**C2**	27.3	27.4	67.2	71.6	210.6	27.9	34.1	24.0	27.9	72.5	138.1	40.2	148.5	140.5
**C3**	78.2	78.3	79.0	76.8	82.7	78.8	217.8	79.8	78.8	79.4	207.1	82.9	80.0	211.9
**C4**	39.3	39.6	38.5	38.7	44.9	38.9	49.0	37.7	38.9	39.3	45.4	145.1	145.2	47.6
**C5**	47.9	53.1	43.1	43.1	49.2	49.8	138.7	49.0	49.3	51.1	49.2	134.5	137.5	79.2
**C6**	24.0	17.9	18.6	18.5	19.1	18.9	144.5	35.3	19.0	20.0	28.5	74.5	73.6	73.6
**C7**	131.7	33.7	30.2	30.1	29.4	29.7	181.4	197.6	29.2	30.4	32.5	41.8	42.1	41.3
**C8**	131.0	47.0	134.8	134.9	126.7	126.8	121.7	125.3	125.0	124.4	124.7	19.3	19.4	18.9
**C9**	164.2	172.7	146.4	146.2	145.8	121.2	152.9	123.6	147.6	149.5	132.3	27.1	27.3	28.6
**C10**	37.3	41.4	38.7	38.8	43.4	37.3	41.0	38.0	37.5	38.3	136.3	17.5	17.2	16.0
**C11**	116.7	122.0	124.2	124.1	110.3	110.6	112.5	104.0	109.7	110.8	117.5	19.0	19.2	19.9
**C12**	202.2	88.7	126.8	126.8	152.2	148.3	162.7	162.6	152.1	154.3	153.0	28.1	28.5	28.3
**C13**	73.1	131.4	135.1	135.2	122.2	151.9	126.8	155.6	119.3	119.3	132.6	38.9	38.1	43.8
**C14**	55.5	150.9	129.8	129.9	131.5	131.1	130.5	129.8	139.1	140.0	130.5	212.8	212.3	210.5
**C15**	138.9	140.0	-	-	15.2	15.3	27.1	16.1	135.1	137.0	22.6	83	87.8	51.5
**C16**	117.9	113.7	-	-	-	-	-	-	119.5	120.4	-	16.9	13.9	10.1
**C17**	14.0	15.2	21.0	21.0	-	-	-	-	12.9	13.3	-	29.7	29.3	26.0
**C18**	27.4	28.3	28.5	28.4	29.2	28.1	21.8	27.5	28.1	30.2	19.8	28.5	28.8	28.9
**C19**	15.5	15.6	21.8	21.9	16.2	15.3	25.2	15.6	15.4	17.3	15.4	14.9	14.9	15.0
**C20**	20.7	21.4	25.8	25.7	25.9	24.8	16.3	23.4	24.9	27.4	122.5	16.9	16.6	13.4
-OCH_3_	-	-	-	-	-	-	-	55.6	-	-	-	-	-	-
-OCOCH_3_	-	-	-	-	-	-	-	170.8	-	-	-	-	-	-
-OCOCH_3_	-	-	-	-	-	-	-	21.2	-	-	-	-	-	-
**Solvent**	CDCl_3_ 125 MHz	CDCl_3_ 125 MHz	CDCl_3_ 100 MHz	CDCl_3_ 100 MHz	CDCl_3_ 100 MHz	CDCl_3_ 125 MHz	CD_3_OD 125 MHZ	CDCl_3_ 125 MHZ	CDCl_3_ 125 MHz	CDCl_3_ 125 MHz	CDCl_3_-CD_3_OD 50 MHz	CDCl_3_ 100 MHz	CDCl_3_ 100 MHz	Pyr-d_5_ N. I
**Reference**	[61]	[61]	[62]	[62]	[63]	[64]	[65]	[64]	[61]	[61]	[66]	[67]	[67]	[68]
**Compound**	**15**	**16**	**17**	**18**	**19**	**20**	**21**	**22**	**23**	**24**	**25**	**26**	**27**	**28**
**C1**	154.3	160.2	151.8	38.6	38.7	38.5	42.3	42.5	155.7	64.0	152.7	36.6	36.4	43.1
**C2**	140.4	140.3	145.4	39.7	39.6	39.7	75.5	75.2	143.6	62.5	142.7	43.6	43.5	75.4
**C3**	210.5	213.2	195.7	87.5	87.7	87.5	208.8	208.4	195.5	195.6	203.4	208.9	208.9	217.3
**C4**	47.8	47.9	139.9	148.3	149.1	148.8	162.2	164.8	137.4	134.3	62.4	167.2	166.3	164.9
**C5**	34.9	33.4	149.9	63.6	63.6	63.6	60.6	61.6	141.9	151.5	57.7	56.3	56.2	55.8
**C6**	71.5	71.6	75.9	65.2	65.4	65.2	63.1	63.8	74.9	75.6	58.6	60.4	60.4	60.8
**C7**	41.0	37.8	41.4	37.9	37.6	37.9	36.9	37.0	42.9	41.9	41.0	40.5	40.5	40.3
**C8**	17.5	20.9	19.6	19.1	19.1	18.8	19.2	19.3	20.5	19.3	18.4	19.5	19.5	19.5
**C9**	27.1	35.8	38.1	35.1	36.2	30.8	36.9	37.3	26.0	26.9	29.1	28.3	28.3	28.3
**C10**	15.7	28.8	26.9	24.6	26.3	30.1	26.8	27.6	17.3	17.7	16.5	18.0	18.0	18.1
**C11**	20.0	28.6	28.9	27.7	27.9	23.9	29.1	29.2	21.5	19.2	18.7	23.5	23.4	23.4
**C12**	28.2	149.5	153.5	137.6	142.8	135.0	145.8	148.4	24.9	28.7	24.3	26.6	26.4	26.5
**C13**	40.3	132.5	128.4	137.9	140.8	138.8	137.8	137.7	30.5	41.2	37.6	40.6	40.3	43.6
**C14**	213.3	199.3	199.0	195.0	194.4	195.2	192.5	192.1	81.5	209.2	214.0	208.3	207.3	207.6
**C15**	85.9	81.8	82.6	141.5	141.9	141.4	136.2	135.2	79.3	82.3	86.4	136.5	135.7	134.1
**C16**	9.9	10.1	10.8	18.2	18.3	18.2	26.1	25.0	10.5	10.1	10.2	17.2	15.7	26.2
**C17**	29.0	29.0	25.5	17.8	17.9	17.8	17.4	17.1	29.5	28.9	18.3	18.5	18.3	18.5
**C18**	28.9	29.1	28.5	29.0	29.0	71.9	29.1	29.9	28.7	28.3	28.2	28.8	28.8	28.8
**C19**	15.1	16.4	15.5	16.1	16.2	11.8	16.4	16.5	14.5	14.9	15.4	15.0	15.0	15.0
**C20**	15.4	11.7	12.0	13.6	58.6	13.8	12.7	12.5	21.3	17.0	14.6	17.8	17.6	17.1
**Solvent**	Pyr-d_5_ 75 MHz	CDCl_3_ 100 MHZ	CDCl_3_ 100 MHz	CDCl_3_ 100 MHz	CDCl_3_ 100 MHz	CDCl_3_ 100 MHz	CDCl_3_ 100 MHz	CDCl_3_ 100 MHz	CDCl_3_ 100 MHz	CDCl_3_ 100 MHz	CDCl_3_ 100 MHz	CDCl_3_ 100 MHz	CDCl_3_-DMSO-d_6_ (9:1) 100 MHz	CDCl_3_ 100 MHz
**Reference**	[69]	[70]	[70]	[70]	[70]	[70]	[70]	[70]	[71]	[72]	[73]	[73]	[73]	[73]
**Compound**	**29**	**30**	**31**	**32**	**33**	**34**	**35**	**36**	**37**	**38**	**39**	**40**	**41**	**42**
**C1**	43.5	150.6	149.5	151.0	149.9	151.5	155.5	42.0	146.6	156.4	151.6	155.3	157.5	154.8
**C2**	75.5	149.2	146.8	145.3	148.7	146.6	143.8	40.4	149.2	144.8	144.9	141.3	139.5	141.5
**C3**	208.8	195.7	196.4	195.4	194.2	195.7	195.7	82.9	194.1	200.9	197.3	197.9	199.7	207.9
**C4**	166.7	132.2	134.6	132.0	133.1	137.0	137.6	145.3	130.4	70.4	134.0	54.7	55.1	54.5
**C5**	56.2	145.5	153.7	145.8	149.0	144.0	141.8	134.6	140.8	75.0	145.0	117.3	120.0	117.9
**C6**	61.0	73.5	73.9	84.4	75.7	76.0	74.6	74.5	75.0	73.9	75.0	146.7	144.7	146.1
**C7**	40.5	43.6	43.5	36.9	41.8	41.5	42.7	41.6	83.1	37.85	42.9	36.9	37.2	36.3
**C8**	19.4	18.1	19.0	18.1	19.0	19.5	20.6	19.5	22.8	20.7	20.5	28.6	28.8	27.8
**C9**	28.6	27.4	24.8	27.1	24.0	26.9	26.2	27.3	25.8	37.88	28.5	34.9	30.4	32.9
**C10**	18.1	17.8	16.9	17.5	17.4	17.9	17.0	17.8	16.3	27.7	17.2	25.6	31.4	30.2
**C11**	23.0	20.1	22.1	20.1	20.9	19.4	21.5	19.3	20.3	28.3	20.9	30.1	26.5	30.7
**C12**	26.1	30.0	28.3	29.5	27.9	29.6	24.8	28.4	27.5	151.3	29.6	152.3	150.0	146.1
**C13**	43.9	38.6	37.4	43.6	38.1	38.2	30.5	38.9	36.6	132.2	38.9	131.9	132.5	134.5
**C14**	207.0	213.5	210.8	212.0	211.1	210.9	81.4	210.6	92.3	195.0	209.8	208.5	208.5	198.1
**C15**	133.2	85.0	84.1	85.3	83.3	84.2	79.8	84.6	68.0	79.2	59.6	84.9	84.9	84.7
**C16**	25.0	10.7	10.8	10.7	10.9	10.9	10.4	17.2	11.2	11.3	11.0	10.7	12.3	10.6
**C17**	18.2	29.2	29.0	23.7	29.1	29.0	29.3	29.8	29.2	20.7	31.2	20.5	20.5	20.4
**C18**	28.7	28.8	28.7	29.0	28.7	28.3	28.5	28.5	28.0	28.5	28.6	29.3	70.8	-
**C19**	15.1	14.9	15.5	14.9	15.7	14.9	14.3	14.6	15.5	16.0	15.3	16.4	12.6	10.2
**C20**	17.1	16.7	19.4	16.5	18.6	16.9	21.4	17.2	11.5	11.6	18.1	11.8	10.5	12.1
-OCH_3_	-	-	-		-	-	-	-	-	-	-	-	-	
-OCOCH_3_	-	-	-	170.1	-	-	-	-	-	-	-	-	-	175.6
-OCOCH_3_	-	-	-	22.1	-	-	-	-	-	-	-	-	-	52.5
**Solvent**	CDCl_3_ 100 MHz	CDCl_3_ 100 MHz	CDCl_3_ 100 MHz	CDCl_3_ 100 MHz	CDCl_3_ 100 MHz	CDCl_3_ 100 MHz	CDCl_3_ 100 MHz	CDCl_3_ 100 MHz	Pyr-d_5_ 150 MHz	CDCl_3_ 150 MHz	Pyr-d_5_ 150 MHz	CDCl_3_ 150 MHz	Pyr-d_5_ 150 MHz	CDCl_3_ 125 Mhz
**Reference**	[73]	[73]	[73]	[73]	[73]	[73]	[74]	[74]	[75]	[75]	[75]	[75]	[75]	[76]
**Compound**	**43**	**44**	**45**	**46**	**47**	**48**	**49**	**50**	**51**	**52**	**53**	**54**	**55**	**56**
**C1**	154.2	152.2	147.6	35.6	159.8	159.8	39.0	N.I	N.I	39.1	39.0	171.9	38.6	171.0
**C2**	140.2	149.4	129.3	40.4	143.8	143.8	75.4	N.I	N.I	76.5	76.4	131.0	203.5	170.4
**C3**	211.1	199.0	194.9	208.2	210.2	210.2	208.5	146.7	164.9	212.9	213.1	60.3	168.1	82.0
**C4**	45.6	61.2	136.1	130.7	53.9	53.9	151.0	N.I	N.I	155.5	155.3	123.8	145.2	121.3
**C5**	89.2	63.5	135.7	57.7	71.8	71.8	44.8	N.I	55.7	70.3	70.7	138.1	55.0	137.6
**C6**	74.0	70.9	74.2	60.4	75.1	75.1	152.7	N.I	n.i	50.2	50.1	73.7	88.8	73.7
**C7**	40.0	84.7	84.8	40.5	41.2	41.2	40.4	41.6	n.i	32.5	32.4	42.2	38.8	45.7
**C8**	17.9	23.5	22.8	19.5	16.1	16.1	26.8	29.7	26.5	19.6	19.9	20.0	20.1	20.1
**C9**	27.7	25.9	27.1	28.6	28.7	28.7	27.1	195.8	207.6	27.6	27.6	27.0	24.9	26.77
**C10**	16.1	16.5	17.6	18.2	17.1	17.1	20.0	N.I	N.I	20.5	20.5	18.4	19.7	18.9
**C11**	19.3	20.1	21.8	23.6	19.4	19.4	27.2	38.2	43.6	22.2	22.2	21.8	27.7	21.8
**C12**	28.1	27.1	35.1	26.8	24.3	24.3	48.6	19.6	19.5	25.1	25.2	28.4	41.1	30.1
**C13**	44.0	37.2	34.2	36.5	38.7	38.7	77.0	27.0	28.2	55.7	55.6	47.7	75.3	44.8
**C14**	209.6	90.9	158.0	69.6	213.5	213.5	203.6	19.5	23.4	203.6	203.4	205.8	202.6	202.2
**C15**	51.1	66.9	116.6	180.5	84.0	84.0	154.9	18.0	18.1	144.7	143.4	157.4	133.5	126.7
**C16**	10.4	11.3	10.8	17.7	10.0	10.0	24.6	17.0	18.5	25.4	25.3	14.7	30.3	13.8
**C17**	25.3	26.8	29.8	29.1	23.9	23.9	111.7	15.0	17.7	13.7	13.8	22.1	20.6	26.80
**C18**	28.9	28.9	29.4	15.3	15.6	15.6	16.5	10.9	15.0	29.0	29.0	29.4	16.4	29.2
**C19**	15.1	15.4	15.8	17.3	28.3	28.3	28.7	29.1	28.8	15.6	15.6	15.6	28.7	15.8
**C20**	13.3	11.9	12.8	13.9	14.9	14.9	21.7	28.4	26.2	17.7	18.0	13.7	21.3	11.8
-OCH_3_	60.3	-	-	-	-	-	-	-	-	-	-	-	-	-
-OCOCH_3_	-	-	-	-	-	171.0	-	-	-	-	-	-	-	-
-OCOCH_3_	-	-	-	-	-	20.9	-	-	-	-	-	-	-	-
**Solvent**	CDCl_3_ 125 MHz	CDCl_3_ 125 MHz	CDCl_3_ 125 MHz	CDCl_3_ 100 MHz	CDCl_3_ 100 MHz	CDCl_3_ 100 MHz	CDCl_3_ 150 MHz	CDCl_3_ 100 MHz	CDCl_3_ 100 MHz	CDCl_3_ 100 MHz	CDCl_3_ 100 MHz	CDCl_3_ 75 MHz	CDCl_3_ 150 MHz	CDCl_3_ 150 MHz
**Reference**	[76]	[76]	[76]	[77]	[77]	[77]	[78]	[79]	[79]	[77]	[77]	[80]	[78]	[75]
**Compound**	**57**	**58**	**59**	**60**	**61**	**62**	**64**	**65**	**66**	**67**	**68**	**69**	**70**	**71**
**C1**	34.4	35.9	152.7	158.8	154.5	154.7	209.6	212.7	212.1	212.3	209.3	204.1	203.3	204.7
**C2**	36.2	37.5	142.7	124.2	146.3	146.2	48.3	39.2	39.8	74.7	73.0	145.7	144.8	49.2
**C3**	74.1	81.8	203.4	131.8	205.5	205.7	76.8	36.3	36.4	43.7	43.1	152.1	152.6	80.3
**C4**	148.0	137.8	62.4	129.4	45.8	46.3	148.4	160.5	158.6	158.1	158.4	88.9	86.3	160.0
**C5**	122.4	135.0	57.7	128.3	209.2	209.0	198.4	199.1	198.5	198.4	197.5	206.3	208.0	209.6
**C6**	79.2	80.7	58.6	114.7	44.2	44.1	141.3	141.2	141.0	141.2	140.9	131.2	136.3	37.3
**C7**	110.1	213.7	41.0	15.8	18.8	18.9	137.2	136.4	136.7	136.9	137.5	139.0	134.4	33.8
**C8**	29.8	37.8	18.4	203.8	26.0	26.1	43.7	43.9	43.8	43.5	43.8	45.7	45.5	45.0
**C9**	21.8	46.6	29.1	40.3	17.8	17.7	45.4	45.6	46.0	45.4	45.6	42.8	43.8	40.6
**C10**	17.7	145.7	16.5	29.0	24.8	24.1	160.5	149.0	149.0	148.7	148.3	57.4	59.1	142.7
**C11**	21.9	138.8	18.7	24.5	29.1	29.5	148.9	147.7	148.8	146.8	146.9	148.7	146.7	147.7
**C12**	25.6	129.8	24.3	17.3	39.8	39.2	36.4	36.7	36.7	36.7	36.4	36.9	36.4	34.2
**C13**	37.9	72.4	37.6	25.9	213.3	213.6	34.3	34.6	34.6	34.6	34.3	34.4	33.7	32.3
**C14**	107.0	176.2	214.0	19.1	82.6	82.7	51.4	51.9	51.9	52.0	51.5	53.7	51.9	48.4
**C15**	47.7	46.7	86.4	44.1	10.5	10.5	146.6	147.0	147.0	145.4	146.0	146.8	150.1	145.4
**C16**	14.2	12.9	10.2	209.8	30.3	30.3	113.4	113.4	113.4	113.6	113.5	112.5	112.5	111.7
**C17**	15.9	27.6	18.3	29.9	29.3	29.4	18.7	18.8	18.8	18.9	18.8	20.2	18.7	17.6
**C18**	29.3	21.5	28.2	17.4	14.8	14.8	108.4	108.2	108.3	108.3	109.1	108.0	107.0	104.7
**C19**	15.1	110.5	15.4	29.0	19.0	18.4	14.5	17.8	14.7	26.3	24.0	10.8	10.6	12.9
**C20**	14.8	19.4	14.6	14.7	-	-	18.9	19.6	19.6	19.6	19.7	22.0	19.0	15.5
-OCH_3_	-	-	-	-	-	-	-	-	-	-	-	-	-	-
-OCOCH_3_	170.8	170.4	-	-	-	-	-	-	-	-	-	-	-	-
-OCOCH_3_	21.0	21.2	-	-	-	-	-	-	-	-	-	-	-	-
**Solvent**	CDCl_3_ 125 MHz	CDCl_3_ 125 MHz	CDCl_3_ 100 MHz	CDCl_3_ 100 MHz	CDCl_3_ 150 MHz	CDCl_3_ 125 MHz	CDCl_3_ 125 MHz	CDCl_3_ 125 MHz	CDCl_3_ 125 MHz	CDCl_3_ 125 MHz	CDCl_3_ 125 MHz	CDCl_3_ 125 MHz	CDCl_3_ 150 MHz	CDCl_3_ 150 MHz
**Reference**	[81]	[81]	[82]	[83]	[84]	[84]	[85]	[86]	[86]	[86]	[86]	[87]	[88]	[88]
**Compound**	**72**	**73**	**74**	**75**	**76**	**77**	**78**	**79**	**80**	**81**	**82**	**83**	**84**	**85**
**C1**	209.9	210.9	210.3	211.9	209.5	208.6	208.3	36.9	104.9	104.0	108.6	108.0	103.8	37.2
**C2**	40.7	40.3	40.4	42.2	74.0	47.1	47.3	37.4	47.7	45.9	38.6	40.8	77.6	37.8
**C3**	34.0	36.0	33.0	31.4	N.I.	72.2	77.8	91.0	80.4	80.3	37.3	74.9	43.9	81.4
**C4**	167.9	153.8	84.1	86.6	157.9	150.7	149.5	140.0	138.3	N.I.	136.1	135.4	138.9	140.1
**C5**	207.7	204.5	217.6	215.4	198.2	197.3	198.0	139.7	192.1	191.5	190.3	192.0	193.6	131.5
**C6**	81.9	64.2	42.1	42.1	142.0	141.7	142.0	48.9	135.6	138.0	138.1	138.2	137.1	49.3
**C7**	70.2	65.5	82.1	80.4	136.0	135.1	134.4	210.4	141.4	140.1	140.0	141.6	138.6	211.7
**C8**	47.1	41.2	47.2	41.6	43.6	43.5	43.7	46.2	37.2	38.4	37.2	37.2	35.3	46.6
**C9**	39.0	43.5	39.5	39.2	45.0	51.7	51.7	46.8	43.4	44.6	43.6	43.5	47.2	210.4
**C10**	140.5	147.6	55.5	51.9	146.8	146.6	146.6	145.9	151.4	149.0	149.5	150.1	147.3	51.6
**C11**	146.8	149.5	146.3	142.8	148.1	34.3	34.4	134.2	75.7	75.7	75.6	75.4	69.8	53.7
**C12**	36.3	36.7	35.3	35.2	36.5	36.5	36.6	131.2	28.8	28.7	25.6	25.6	32.2	131.9
**C13**	34.0	35.6	33.3	33.5	34.3	148.2	148.0	53.4	25.6	27.8	28.7	28.8	25.7	134.6
**C14**	47.5	51.9	47.9	48.1	51.9	45.3	45.0	211.0	49.0	49.5	48.8	49.0	51.3	48.9
**C15**	146.7	146.6	146.0	146.4	146.5	156.0	157.4	51.2	145.8	146.0	146.0	145.8	146.5	146.3
**C16**	112.9	114.3	112.5	112.4	113.3	8.4	15.3	13.6	114.1	114.5	113.9	114.1	113.3	110.5
**C17**	19.1	19.6	18.2	18.5	18.6	18.8	18.5	16.8	20.4	19.2	18.9	19.0	18.9	21.7
**C18**	106.8	108.4	106.5	109.0	108.3	113.3	113.3	21.3	73.4	68.8	73.3	73.7	70.0	17.1
**C19**	18.2	14.8	14.5	18.0	24.0	18.6	18.6	110.1	19.0	10.1	11.0	7.7	23.6	14.0
**C20**	26.8	17.3	15.2	15.6	18.7	108.4	108.2	17.7	9.0	20.8	20.8	20.5	20.5	18.1
-OCH_3_	-	-	-	-	-	-	-	-	-	-	-	-	-	-
-OCOCH_3_	-	-	-	-	-	169.9	170.1	170.8	-	-	-	-	-	171.2
-OCOCH_3_	-	-	-	-	-	20.4	20.6	21.3	-	-	-	-	-	21.6
**Solvent**	CDCl_3_ 150 MHz	CDCl_3_ 150 MHz	CDCl_3_ 150 MHz	CDCl_3_ 150 MHz	CDCl_3_ 100 MHz	CDCl_3_ 100 MHz	CDCl_3_ 100 MHz	CDCl_3_ 125 MHz	CDCl_3_ 100 MHz	CDCl_3_ 100 MHz	CDCl_3_ 150 MHz	CDCl_3_ 100 MHz	CDCl_3_ 150 MHz	CDCl_3_ 100 MHz
**Reference**	[88]	[88]	[88]	[88]	[89]	[77]	[77]	[90]	[91]	[92]	[88]	[92]	[93]	[92]
**Compound**	**86**	**87**	**88**	**89**	**90**	**91**	**92**	**93**	**93**	**94**	**95**	**96**	**97**	**98**
**C1**	169.1	180.6	207.0	210.3	209.9	199.6	194.3	208.8	208.7	207.4	206.2	158.7	158.6	157.8
**C2**	205.2	35.0	38.8	39.3	40.2	36.8	153.1	144.3	144.2	59.2	58.7	112.8	120.2	119.4
**C3**	42.5	42.3	41.6	38.1	38.4	38.4	132.1	153.4	153.6	53.7	52.7	141.8	146.7	145.1
**C4**	142.6	205.1	200.3	142.7	143.1	190.0	201.2	33.0	33.1	144.7	136.2	135.2	138.6	138.9
**C5**	193.6	167.2	170.9	167.5	167.5	169.0	79.3	199.7	199.5	203.1	200.7	30.6	23.2	24.1
**C6**	137.0	126.2	127.4	147.7	147.6	127.1	133.6	133.5	133.4	142.9	142.8	26.7	26.9	25.9
**C7**	145.0	138.1	147.5	118.0	118.1	144.2	131.2	146.6	146.8	130.2	129.8	126.9	125.6	125.9
**C8**	40.9	45.4	44.9	46.3	46.5	43.1	39.3	40.9	41.1	44.0	43.9	135.2	132.3	130.9
**C9**	46.9	43.3	43.9	41.7	41.6	42.5	41.2	47.9	48.1	46.0	45.5	41.5	47.2	46.0
**C10**	137.5	129.4	114.2	153.6	152.8	114.7	55.4	60.7	60.7	162.8	162.5	129.1	75.5	75.6
**C11**	83.3	145.6	146.6	146.8	146.7	144.2	146.9	145.3	145.6	148.7	147.8	126.8	129.3	124.9
**C12**	28.3	35.3	36.8	35.5	35.5	35.4	35.6	36.8	37.0	36.6	36.4	135.5	136.4	137.3
**C13**	25.9	32.9	32.7	33.2	33.1	31.6	33.0	33.6	33.8	34.3	34.3	123.0	38.6	34.4
**C14**	46.4	51.8	54.5	51.4	51.4	52.4	50.7	50.4	50.6	51.5	51.4	27.6	28.4	26.7
**C15**	145.6	146.7	150.4	147.1	147.1	148.5	146.8	146.2	146.3	146.9	146.7	33.7	33.3	29.9
**C16**	114.6	112.4	111.1	112.0	112.0	111.1	16.7	113.4	113.5	113.3	113.0	22.1	23.3	21.4
**C17**	18.8	18.6	19.6	18.5	18.5	19.3	19.0	19.4	19.4	18.7	18.4	22.1	20.8	21.2
**C18**	69.4	108.1	108.6	107.9	108.1	108.2	19.3	106.7	106.9	21.1	19.7	190.7	195.1	195.4
**C19**	30.2	17.8	19.0	17.3	15.7	17.8	112.2	10.5	10.5	17.9	17.9	16.0	15.9	16.3
**C20**	21.5	18.9	21.5	19.8	19.7	21.6	106.0	16.1	16.2	108.2	108.5	20.2	16.4	18.0
-O**C**H_3_	-	-	-	-	-	-	-	-	-	-	-	-	55.3	55.5
**-OCO**CH_3_	-	-	-	-	-	-	-	-	-	-	-	-	-	-
**-**OCO**CH_3_**	-	-	-	-	-	-	-	-	-	-	-	-	-	-
**Solvent**	CDCl_3_ 150 MHz	CDCl_3_ 150 MHz	MeOD-d_4_ 150 MHz	CDCl_3_ 150 MHz	CDCl_3_ 150 MHZ	DMSO 1% TFA-d 125 MHz	CDCl_3_ 150 MHz	CDCl_3_ 125 MHz	CDCl_3_ 125 MHz	CDCl_3_ 150 MHz	CDCl_3_ 150 MHz	CDCl_3_ 125 MHz	CDCl_3_ 125 MHz	CDCl_3_ 125 MHz
**Reference**	[88]	[88]	[88]	[88]	[88]	[94]	[78]	[87]	[86]	[95]	[95]	[96]	[96]	[96]
														
**Compound**	**99**	**100**	**101**	**102**	**103**	**104**	**105**	**106**	**107**	**108**	**109**	**110**	**111**	**112**
**C1**	42.5	49.9	49.4	48.6	153.8	150.3	216.9	218.0	30.3	30.1	208.2	208.2	30.3	30.5
**C2**	38.3	37.8	81.2	80.8	143.9	144.7	213.8	215.6	42.5	42.4	42.5	42.6	42.2	40.0
**C3**	123.7	154.7	122.8	122.5	196.7	195.2	207.2	210.6	207.7	208.1	30.2	30.1	208.4	208.0
**C4**	137.1	138.8	137.3	140.3	136.0	132.5	144.7	144.5	136.9	137.5	134.3	134.3	146.0	145.5
**C5**	147.0	131.3	147.6	146.0	139.5	139.7	129.1	138.4	134.3	134.4	146.1	150.1	133.1	133.0
**C6**	141.8	136.8	142.8	142.9	82.8	81.6	72.5	135.9	145.5	145.0	114.8	114.8	145.5	145.5
**C7**	201.8	202.9	201.3	201.3	37.6	37.6	67.8	128.1	33.4	33.3	137.4	137.1	33.2	33.3
**C8**	128.7	66.7	128.7	128.7	25.6	25.5	65.6	64.5	20.8	20.8	25.9	25.9	21.1	21.1
**C9**	158.7	79.9	159.1	159.9	88.5	88.1	55.5	55.5	23.1	23.1	16.1	16.1	25.5	25.5
**C10**	36.6	37.0	36.7	37.0	36.0	35.7	52.9	51.4	23.0	23.0	19.4	19.5	19.0	19.0
**C11**	41.3	43.3	41.3	41.6	34.5	34.4	46.8	51.0	25.9	25.8	15.8	15.8	27.8	27.8
**C12**	183.1	76.9	183.9	183.4	31.3	30.9	38.0	39.8	135.4	135.3	33.4	33.5	136.7	136.7
**C13**	112.4	87.7	112.4	113.0	42.0	43.5	37.0	38.3	130.6	131.0	150.1	42.4	131.7	131.3
**C14**	203.8	213.7	203.2	203.4	211.8	204.6	35.0	38.0	150.1	150.3	131.1	131.8	150.7	150.6
**C15**	99.8	91.4	97.7	97.9	82.4	88.9	34.2	37.4	132.7	131.8	145.5	145.6	130.5	130.5
**C16**	18.9	19.2	26.9	27.1	10.6	10.7	27.7	27.9	15.8	17.0	13.2	13.3	16.7	15.5
**C17**	20.7	21.9	20.7	21.1	24.2	24.0	23.2	23.6	115.2	115.5	115.8	28.2	114.5	114.2
**C18**	30.4	27.5	30.2	30.5	24.1	28.2	20.2	20.7	73.8	73.8	17.1	17.1	28.0	28.0
**C19**	26.9	22.0	26.7	27.1	28.4	23.4	16.2	20.5	12.1	12.1	17.3	21.4	15.8	15.8
**C20**	6.0	14.2	5.9	6.3	20.6	20.6	14.7	14.0	12.6	12.6	13.2		13.3	13.3
-OCH_3_	-	-	-	-	-	-	-	-	-	-	-	-	-	-
-OCOCH_3_	-	-	-	-	-	169.3	-	-	171.3	171.3	-	-	-	-
-OCOCH_3_	-	-	-	-	-	22.1	-	-	20.9	20.9	-	-	-	-
**Solvent**	CDCl_3_ 62,9 MHz	CDCl_3_ 100 MHz	CDCl_3_ 62,9 MHz	CDCl_3_ 125 MHz	CDCl_3_ 90/150 MHz	CDC_l3_ 100 MHz	C_6_D_6_ 150 MHz	CDCl_3_ 125 MHz	CDCl_3_ 100 MHz	CDCl_3_ 100 MHz	CDCl_3_ 75 MHz	CDCl_3_ 100 MHz	CDCl_3_ 50 MHz	CDCl_3_ 50 MHz
**Reference**	[97]	[98]	[97]	[99]	[72]	[100]	[101]	[96]	[97]	[98]	[97]	[99]	[72]	[100]
**Compound**	**113**	**114**	**115**	**116**	**117**	**118**	**119**	**120**	**121**	**122**	**123**	**124**	**125**	**125**
**C1**	30.3	30.2	30.0	37.6	37.2	30.6	11.9	74.0	67.1	30.5	30.2	30.4	40.5	41.6
**C2**	42.4	42.5	42.3	77.7	77.5	42.1	13.3	50.2	45.9	42.1	42.4	42.7	38.0	39.3
**C3**	208.5	207.6	207.8	207.1	206.1	206.2	16.9	207.5	209.0	206.4	207.6	208.0	32.6	33.5
**C4**	139.7	137.2	137.7	134.3	134.6	136.9	20.9	157.4	157.1	137.9	138.1	137.5	149.7	150.3
**C5**	133.9	134.4	134.4	135.3	135.7	133.0	22.0	45.6	45.8	134.1	134.3	134.0	203.4	206.3
**C6**	145.4	144.7	144.2	146.0	144.4	146.3	25.4	153.5	153.7	143.9	144.0	145.5	48.0	49.5
**C7**	33.3	33.5	33.5	33.3	33.4	33.7	24.7	40.1	40.1	33.6	33.7	33.55	52.6	52.9
**C8**	21.3	20.7	20.7	21.4	21.4	20.9	30.7	27.0	27.0	20.5	20.5	N.I.	181.3	184.8
**C9**	26.2	28.0	28.0	26.0	25.9	22.1	33.5	26.5	26.7	25.9	26.0	26.2	65.5	67.3
**C10**	19.6	26.7	26.7	19.6	19.6	25.0	41.9	19.5	19.5	35.8	35.8	23.3	206.8	208.8
**C11**	27.3	30.4	30.4	28.3	28.3	25.5	70.5	35.3	35.3	28.5	28.6	23.1	33.2	34.1
**C12**	134.7	134.2	134.3	138.1	137.9	136.0	114.5	47.1	46.9	132.9	133.0	136.0	22.73	23.7
**C13**	130.0	130.1	130.3	132.1	132.4	132.6	131.1	49.2	49.5	130.1	130.2	131.0	35.9	36.8
**C14**	153.2	150.1	150.1	150.3	150.1	151.3	132.3	202.3	201.8	150.4	150.4	150.6	207.1	209.2
**C15**	133.3	132.9	132.1	129.7	128.7	131.4	132.8	152.8	153.7	132.2	132.4	131.8	26.2	27.1
**C16**	17.0	15.8	17.0	26.1	25.7	15.0	135.7	14.2	9.0	15.0	16.4	17.3	28.3	28.4
**C17**	115.3	116.0	116.3	115.1	116.2	113.9	138.0	110.3	110.3	115.8	116.5	115.6	16.7	17.2
**C18**	28.0	176.2	176.2	28.1	28.1	70.7	145.5	16.8	16.8	202.3	202.4	12.5	12.4	11.9
**C19**	16.5	10.3	10.2	16.1	16.2	11.4	151.3	28.4	28.4	7.8	7.9	74.1	21.5	22.0
**C20**	62.1	13.0	13.0	13.3	13.4	12.7	207.6	11.4	11.1	12.8	12.8	13.2	17.6	17.8
-O**C**H_3_	-	52.3	52.3	-	-	-	-	-	-	-	-	-	-	-
**-OCO**CH_3_	-	-	-	-	-	-	-	-	-	-	-	170.5	-	-
**-**OCO**CH_3_**	-	-	-	-	-	-	-	-	-	-	-	10.9	-	-
**Solvent**	CDCl_3_ 150 MHz	CDCl_3_ 150 MHz	CDCl_3_ 150 MHz	CDCl_3_ 100 MHz	CDCl_3_ 100 MHz	CDCl_3_ 150 MHz	DMSO-d_6_ 100 MHz	CDCl_3_ 100 MHz	CDCl_3_ 150 MHz	CDCl_3_ 150 MHz	CDCl_3_ 150 MHz	CDCl_3_ 125 MHz	CDCl_3_ 100 MHz	MeOD-d_4_ 100 MHz
**Reference**	[102]	[102]	[102]	[89]	[89]	[103]	[104]	[77]	[77]	[103]	[103]	[87]	[105]	[105]
														
**Compound**	**126**	**126**	**127**	**128**	**129**	**130**	**131**	**132**	**133**	**134**				
**C1**	40.4	41.5	150.9	27.3	27.8	34.3	34.3	75.7	77.0	31.6				
**C2**	37.8	39.1	144.6	41.6	42.8	39.7	39.5	51.0	48.3	41.5				
**C3**	32.7	33.6	195.2	207.3	205.3	213.1	213.1	209.2	207.9	214.8				
**C4**	149.3	150.0	129.8	128.7	129.5	142.0	142.0	142.3	145.3	157.6				
**C5**	203.3	206.6	149.3	148.5	147.9	33.9	34.0	35.5	35.3	71.0				
**C6**	50.3	51.8	39.4	38.4	38.1	38.5	38.4	39.8	39.3	48.0				
**C7**	45.6	46.9	37.9	38.0	38.4	38.1	38.0	39.3	39.0	30.4				
**C8**	180.6	184.1	16.0	16.0	16.1	16.4	16.4	24.3	24.0	21.2				
**C9**	68.6	70.2	19.2	19.3	19.4	24.0	23.8	157.7	158.0	37.1				
**C10**	205.3	207.5	17.4	17.3	17.1	21.7	21.7	36.7	36.5	44.0				
**C11**	33.2	34.5	20.9	20.4	20.0	29.9	29.5	121.8	121.3	73.7				
**C12**	21.8	23.3	40.9	42.1	42.1	157.7	157.7	155.8	155.7	45.3				
**C13**	37.8	38.0	81.7	81.8	81.7	137.2	137.2	132.1	131.6	54.0				
**C14**	206.8	209.5	208.3	208.5	209.1	192.3	192.5	191.3	188.6	204.9				
**C15**	26.3	27.4	81.3	43.1	44.1	161.3	161.3	163.4	158.4	147.6				
**C16**	28.4	28.6	10.8	16.4	14.2	16.4	16.3	14.3	14.7	16.1				
**C17**	16.5	16.9	19.4	19.4	18.9	24.6	24.4	20.7	20.7	14.3				
**C18**	10.7	11.0	15.7	28.6	28.8	28.6	28.7	21.9	21.2	28.3				
**C19**	21.5	22.1	28.5	15.7	15.7	16.1	16.1	21.6	21.4	15.1				
**C20**	11.8	12.1	27.7	26.9	27.0	17.0	16.9	16.2	16.0	14.0				
-OCH_3_	-	-	-	-	-	-	-	-	-	-				
-OCOCH_3_	-	-	-	-	-	-	-	-	170.8	-				
-OCOCH_3_	-	-	-	-	-	-	-	-	20.8	-				
**Solvent**	CDCl_3_ 100 MHz	MeOD-d_4_ 100 MHz	CDCl_3_ 125 MHz	CDCl_3_ 150 MHz	CDCl_3_ 150 MHz	CDCl_3_ 125 MHz	CDCl_3_ 125 MHz	Pyr-d_5_ 100 MHz	Pyr-d_5_ 150 MHz	CDCl_3_ 125 MHz				
**Reference**	[105]	[105]	[75]	[106]	[106]	[106]	[106]	[107]	[107]	[108]				
**Compound**	**135**				**136**				**137**					
	**C1**	161.2	**C1’**	173.3	**C1**	161.2	**C1’**	173.7	**C1**	160.8	**C1’**	173.8		
	**C2**	133.4	**C2’**	38.1	**C2**	133.0	**C2’**	38.1	**C2**	133.1	**C2’**	38.6		
	**C3**	208.7	**C3’**	125.0	**C3**	208.8	**C3’**	121.8	**C3**	208.7	**C3’**	123.3		
	**C4**	74.0	**C4’**	136.8	**C4**	73.6	**C4’**	135.9	**C4**	74.0	**C4’**	133.9		
	**C5**	39.1	**C5’**	51.7	**C5**	38.8	**C5’**	132.6	**C5**	38.9	**C5’**	129.8		
	**C6**	140.9	**C6’**	45.0	**C6**	140.4	**C6’**	128.9	**C6**	140.7	**C6’**	131.6		
	**C7**	129.5	**C7’**	133.0	**C7**	129.4	**C7’**	47.0	**C7**	129.1	**C7’**	48.9		
	**C8**	38.9	**C8’**	131.0	**C8**	38.3	**C8’**	49.2	**C8**	38.5	**C8’**	47.1		
	**C9**	76.4	**C9’**	133.1	**C9**	75.9	**C9’**	139.4	**C9**	76.0	**C9’**	137.7		
	**C10**	56.1	**C10’**	118.1	**C10**	55.7	**C10’**	115.8	**C10**	55.9	**C10’**	115.9		
	**C11**	36.7	**C11’**	175.2	**C11**	36.3	**C11’**	174.8	**C11**	36.5	**C11’**	166.6		
	**C12**	32.0	**C12’**	51.1	**C12**	31.7	**C12’**	53.6	**C12**	31.9	**C12’**	119.0		
	**C13**	63.4	**C13’**	26.8	**C13**	64.3	**C13’**	30.0	**C13**	64.4	**C13’**	145.9		
	**C14**	33.4	**C14’**	32.3	**C14**	30.0	**C14’**	35.3	**C14**	30.2	**C14’**	42.1		
	**C15**	25.9	**C15’**	23.3	**C15**	26.1	**C15’**	24.4	**C15**	26.3	**C15’**	44.1		
	**C16**	74.0	**C16’**	130.0	**C16**	68.7	**C16’**	134.1	**C16**	69.4	**C16’**	132.0		
	**C17**	12.0	**C17’**	134.9	**C17**	10.6	**C17’**	128.8	**C17**	10.9	**C17’**	130.4 or 132.7		
	**C18**	18.7	**C18’**	130.8	**C18**	18.4	**C18’**	130.7	**C18**	18.6	**C18’**	130.4 or 132.8		
	**C19**	10.1	**C19’**	131.8	**C19**	10.2	**C19’**	130.7	**C19**	10.2	**C19’**	130.4 or 132.9		
	**C20**	68.4	**C20’**	131.0	**C20**	68.2	**C20’**	130.7	**C20**	68.2	**C20’**	130.7		
			**C21’**	135.8			**C21’**	134.6			**C21’**	135.6		
			**C22’**	35.3			**C22’**	35.0			**C22’**	35.3		
			**C23’**	22.9			**C23’**	22.6			**C23’**	23.0		
			**C24’**	13.9			**C24’**	14.0			**C24’**	14.0		
**Solvent**	CDCl_3_/125 MHz	CDCl_3_ 125 MHz	CDCl_3_ 125 MHz	35.3			**C22’**	35.3			**C22’**	35.3		
				22.8			**C23’**	22.8			**C23’**	23.0		
			**C24’**	13.9			**C24’**	13.9			**C24’**	14.0		
**Reference**	[109]	[109]	[109]											
**Compound**	**138**				**139**					**140**				
	**C1**	161.0	**C1’**	173.5	**C1**	161.0		**C1’**	173.5	**C1**	161.0	**C1’**	173.9	
	**C2**	133.3	**C2’**	39.0	**C2**	133.3		**C2’**	39.0	**C2**	133.2	**C2’**	39.0	
	**C3**	208.9	**C3’**	122.0	**C3**	208.9		**C3’**	122.3	**C3**	208.7	**C3’**	122.1	
	**C4**	74.2	**C4’**	136.6	**C4**	74.2		**C4’**	136.4	**C4**	74.2	**C4’**	136.7	
	**C5**	38.8	**C5’**	49.9	**C5**	38.8		**C5’**	51.7	**C5**	38.9	**C5’**	129.9–132.3	
	**C6**	140.8	**C6’**	28.6	**C6**	140.8		**C6’**	26.8	**C6**	140.9	**C6’**	130.6	
	**C7**	129.3	**C7’**	31.7	**C7**	129.3		**C7’**	29.6	**C7**	129.4	**C7’**	129.9–132.3	
	**C8**	38.8	**C8’**	22.1	**C8**	38.8		**C8’**	24.1	**C8**	38.6	**C8’**	135.1	
	**C9**	76.2	**C9’**	139.8	**C9**	76.2		**C9’**	135.1	**C9**	76.0	**C9’**	35.7	
	**C10**	55.9	**C10’**	112.4	**C10**	55.9		**C10’**	118.7	**C10**	56.1	**C10’**	31.9	
	**C11**	36.5	**C11’**	173.9	**C11**	36.5		**C11’**	174.6	**C11**	36.6	**C11’**	166.3	
	**C12**	31.9	**C12’**	52.0	**C12**	31.9		**C12’**	54.7	**C12**	31.9	**C12’**	119.6	
	**C13**	63.4	**C13’**	45.4	**C13**	63.4		**C13’**	44.3	**C13**	64.6	**C13’**	153.3	
	**C14**	33.2	**C14’**	134.8	**C14**	33.2		**C14’**	135.1	**C14**	30.3	**C14’**	46.1	
	**C15**	25.5	**C15’**	130.3	**C15**	25.5		**C15’**	130.3	**C15**	26.6	**C15’**	43.4	
	**C16**	73.9	**C16’**	129–134	**C16**	73.9		**C16’**	129–134	**C16**	69.3	**C16’**	136.4	
	**C17**	12.1	**C17’**	129–134	**C17**	12.1		**C17’**	129–134	**C17**	11.0	**C17’**	129.9–132.3	
	**C18**	18.5	**C18’**	129–134	**C18**	18.5		**C18’**	129–134	**C18**	18.6	**C18’**	129.9–132.3	
	**C19**	10.2	**C19’**	129–134	**C19**	10.2		**C19’**	129–134	**C19**	10.4	**C19’**	129.9–132.3	
	**C20**	68.2	**C20’**	131.1	**C20**	68.2		**C20’**	131.1	**C20**	68.2	**C20’**	130.9	
			**C21’**	135.9				**C21’**	135.9			**C21’**	135.3	
			**C22’**	35.3				**C22’**	35.3			**C22’**	35.3	
			**C23’**	22.8				**C23’**	22.8			**C23’**	23.0	
			**C24’**	13.9				**C24’**	13.9			**C24’**	14.0	
**Solvent**	CDCl_3_ / 125 MHz				CDCl_3_ 125 MHz					CDCl_3_ 125 MHz				
**Reference**	[109]				[109]					[109]				

Not informed (N.I).

**Table 2:** Top ten JDT with remarkable cytotoxic activity.

Compound	IC_50_ (Cell Lineage) *	Assay **	Reference
Curcusone C	0.08 µM (HEPG2) 0.25 µM (L5178y)	MTT	[88,133]
Curcusone D	0.15 µM (HEPG2) 0.51 µM (L5178y)	MTT	[88,133]
Curcusone B	0.70 µM (L5178y) 2.64 µM (HL-60)	MTT	
Curcusone A	0.91 µM (L5178y) 1.63 µM (HL-60)	MTT	
Jatrophone	1.80 µM (MCF-7) 1.82 µM (U-251)	SRB	[134,135]
Jatromultone D	2.69 µM (A549)	MTT	[77]
Jatrogrossidione	2.6 µM (RKO)	CKK-8	[136]
15-*epi*-4Z- Jatrogrossidentadion	2.61 µM (HEPG2)	MTT	[77]
2-*epi*-hydroxyiso Jatrogrossidion	2.67 µM (HEPG2) 3.20 µM (HL-60) 3.20 µM (MCF-7)	MTS	[77]
3-dehydroxy-2-*epi*-caniojane	2.86 µM (HL-60)	MTT	[88]

* Cell lineage: HEPG2—human hepatocarcinoma; L5178y—murine leukemia; HL-60—human leukemia; MCF-7—breast cancer; U-251—human malignant glioblastoma; A549—human lung carcinoma; RKO—human colorectal carcinoma. ** Assay: MTT—(3-(4, 5-dimethylthiazolyl-2)-2, 5-diphenylte-trazolium bromide); MTS—(3-(4,5-dimethylthiazol-2-yl)-5-(3-carboxymethoxyphenyl)-2-(4-sulfo-phenyl)-2H-tetrazolium); SBR—Sulforhodamine B; CKK-8—Cell Counting Kit-8.

## Data Availability

Not applicable.

## Supplementary Materials

The following supporting information can be downloaded at https://www.mdpi.com/article/10.3390/ph17101399/s1: Table S1. List of biological activities of isolated diterpenes from *Jatropha* species; Expansions of Figure 5: ADMET and Drug-likeness; Refs [175,176,177,178,179,180,181,182,183,184,185,186,187,188,189,190,191,192,193,194,195,196,197,198,199,200,201,202,203,204,205,206,207,208,209,210,211,212,213,214] are mentioned in Supplementary materials.
