# Supplementary material for: Jatropha Diterpenes: An Updated Review Concerning Their Structural Diversity, Therapeutic Performance, and Future Pharmaceutical Applications
Source: Pharmaceuticals (Basel). 2024 Oct 19;17(10):1399. doi: 10.3390/ph17101399 (PMC11510188; doi:10.3390/ph17101399)

Jatropha Diterpenes: An Updated Review Concerning Their Structural Diversity, Therapeutic Performance and Future Pharmaceutical Applications

Thalisson A. de Souza [1], Luiz H. A. Pereira [1], Alan F. Alves [2], Douglas Dourado [3], Jociano S. Lins [1], Marcus T. Scotti [2], Luciana Scotti [2], Lucas S. Abreu [4], Josean F. Tavares [1], Marcelo S. da Silva [1,*]

[1] Universidade Federal da Paraíba, Multi-user Characterization and Analysis Laboratory, Research Institute for Drugs and Medicines (IpeFarM), João Pessoa-PB, Brazil.

[2] Universidade Federal da Paraíba, Laboratory of Cheminformatics, Program of Post-graduation on Natural and Synthetic Bioactive Products (PgPNSB), Health Sciences Center, João Pessoa-PB, Brazil.

[3] Instituto Aggeú Magalhães, Department of Imunology, FIOCEUZ-PE.

[4] Universidade Federal Fluminense, Department of Organic Chemistry, Niterói-RJ, Brazil.

* Correspondence: marcelosobral@ltf.ufpb.br

Biological activity

Table S1. List of biological activities of isolated diterpenes from *Jatropha* species.

Name of the compound	Biological activity	Reference
**Jatrogrossidione**	Leishmanicidal	[175]
	Trypanocidal	[175]
	Cytotoxicity	[89]
	Cytotoxicity/Apoptosis induction	[136]
	Thioredoxin reductase inhibition	[71]
	Anti-inflammatory/Inhibition of Inhibition of NO production	[176]
**2-epi-jatrogrossidione**	Cytotoxicity	[89]
	Thioredoxin reductase inhibition	[71]
	Anti-inflammatory/Inhibition of NO production	[176]
	Antimicrobial	[86]
**Caniojane**	Cytotoxicity	[106]
	Antiplasmodial	[90]
	Antituberculosis	[90]
	Cytotoxicity	[90]
**1,11-bisepicaniojane**	Antiplasmodial	[90]
	Antituberculosis	[90]
	Cytotoxicity	[90]
**Jatrogrossidion**	Antiherpes (HSV-1)	[177]
	Cytotoxicity	[76]
	Antiproliferative	[78]
**Isojatrogrossidion**	Cytotoxicity	[76]
**2-epi-isojatrogrossidion**	Cytotoxicity	[76]
**2-epi-hydroxyisojatrogrossidion**	Cytotoxicity	[76]
	Cytotoxicity	[133]
	Antimicrobial	[73]
	Antiherpes (HSV-1)	[177]
	Antiproliferative	[78]
	Cytotoxicity	[78]
**2-hydroxyisojatrogrossidion**	Cytotoxicity	[133]
	Cytotoxicity	[76]
	Antimicrobial	[73]
	Antiherpes (HSV-1)	[177]
	Antiproliferative	[78]
**Jatrophalactone**	Cytotoxicity	[79]
	Cytotoxicity	[178]
**Jatrophalone**	Cytotoxicity	[79]
	Cytotoxicity	[178]
**Jatrophadiketone**	Analgesic	[179]
	Anti-inflammatory	[179]
	Antipyretic	[179]
	NO elimination	[179]
**Curcusone A**	Cytotoxicity	[89]
	Cytotoxicity	[133]
	Thioredoxin reductase inhibition	[71]
**Curcusone B**	Cytotoxicity	[89]

	Cytotoxicity	[133]
	Thioredoxin reductase inhibition	[71]
	Antimetastatic	[180]
	Antiproliferative	[181]
	Cytotoxicity	[182]
**Curcusone C**	Cytotoxicity	[89]
	Cytotoxicity	[133]
	Thioredoxin reductase inhibition	[71]
	Cytotoxicity	[103]
	Antiproliferative and apoptotic	[141]
	Antitumoral - prostate cancer	[142]
**Curcusone D**	Cytotoxicity	[89]
	Cytotoxicity	[133]
	Thioredoxin reductase inhibition	[71]
	Cytotoxicity	[141]
	Antitumoral	[144]
**Curcusone E**	Cytotoxicity	[133]
	Cytotoxicity	[89]
**Curcusone F**	Cytotoxicity	[89]
**Curcusone G**	Cytotoxicity	[89]
**Curcusone H**	Cytotoxicity	[89]
**Curcusone I**	Cytotoxicity	[89]
**Curcusone J**	Cytotoxicity	[89]
**3-dehydroxy-2-epi-caniojane**	Cytotoxicity	[89]
**4-epi-curcusone E**	Cytotoxicity	[89]
**4Z-jatrogrossidentadione**	Antibacterial	[133]
	Antiherpes (HSV-1)	[177]
	Cytotoxicity	[133]
	Cytotoxicity	[76]
	Cytotoxicity	[78]
**4E-jatrogrossidentadione**	Antiherpes (HSV-1)	[177]
	Cytotoxicity	[103]
	Cytotoxicity	[133]
	Cytotoxicity	[76]
	Cytotoxicity	[78]
**15-epi-4E-jatrogrossidentadion**	Antiherpes (HSV-1)	[177]
	Cytotoxicity	[133]
	Cytotoxicity	[76]
	Cytotoxicity	[78]
	Cytotoxicity	[103]
**15-epi-4Z-jatrogrossidentadion**	Antiherpes (HSV-1)	[177]
	Cytotoxicity	[78]
	Antibacterial	[73]
	Cytotoxicity	[76]
**Japodagrin**	Antibacterial	[73]
**Multidione**	Cytotoxicity	[133]
**Multifidanol**	Cytotoxicity	[68]
	Antimicrobial	[68]
**Multifidenol**	Cytotoxicity	[68]
	Antimicrobial	[68]
**Multifidone**	Cytotoxicity	[83]
**14-deoxy-1β-hydroxy-4(4E)-jatrogrossidentadione**	Antimalarial	[75]
	Antimicrobial	[75]

	Antileishmanial	[75]
**15-deoxy-1β-hydroxy-4(4E)-jatrogrossidentadione**	Antimalarial	[75]
	Antimicrobial	[75]
	Antileishmanial	[75]
**Jatrocurcasenone A**	Cytotoxicity	[76]
**Jatrocurcasenone B**	Cytotoxicity	[76]
**Jatrocurcasenone C**	Cytotoxicity	[76]
**Jatrocurcasenone D**	Cytotoxicity	[76]
**Jatrocurcasenone E**	Cytotoxicity	[76]
**Jatrocurcasenone F**	Anti-inflammatory	[77]
**Jatrocurcasenone G**	Anti-inflammatory	[77]
**Jatrocurcasenone H**	Anti-inflammatory	[77]
**Jatrocurcasenone I**	Anti-inflammatory	[77]
**Japodagricanone A**	Cytotoxicity	[85]
**Japodagricanone B**	Cytotoxicity	[85]
	Antimicrobial	[85]
**Jatrointelone A**	Thioredoxin reductase inhibition	[71]
	Thioredoxin reductase inhibition	[64]
**Jatrointelone B**	Thioredoxin reductase inhibition	[71]
**Jatrointelone C**	Thioredoxin reductase inhibition	[71]
**Jatrointelone D**	Thioredoxin reductase inhibition	[71]
**Jatrointelone E**	Thioredoxin reductase inhibition	[71]
**Jatrointelone F**	Thioredoxin reductase inhibition	[71]
**Jatrointelone I**	Thioredoxin reductase inhibition	[71]
**Jatropha factor C1**	Effect on platelet aggregation	[183]
**Jatropha factor C2**	Effect on platelet aggregation	[183]
**Jatropha factor C3**	Effect on platelet aggregation	[183]
**Jatropha factor C4**	Effect on platelet aggregation	[183]
**Jatropha factor C5**	Effect on platelet aggregation	[183]
**Jatrophenone**	Antimicrobial	[91]
**Jatrophone**	Leishmanicidal	[175]
	Insulin release inhibition	[184]
	Uterine muscle contraction inhibition	[159]
	Effect on smooth and cardiac muscle	[157]
	Portal vein contraction inhibition	[158]
	Molluscicidal	[185]
	Vasorelaxant in isolated rat aorta	[186]
	Cytotoxicity	[134]
	Lymphocyte activation inhibition	[187]
	Platelet aggregation inhibition	[188]
	Antiproliferative and antimigratory	[189]
	Gastroprotective	[99]

	Glutamate and GMP-PNP binding inhibition	[190]
	Antiviral and anti-inflammatory	[191]
	Antiproliferative	[192]
	Cytotoxicity	[189]
	Cytotoxicity	[135]
**Japodagrone**	Antibacterial	[91]
**9β,13α-dihydroxyisabellione**	Gastroprotective	[99]
	Antiproliferative	[192]
**Jatropholone A**	Molluscicidal	[185]
	Gastroprotective	[99]
	Antiplasmodial	[90]
	Antituberculosis	[90]
	Cytotoxicity	[90]
	Cytotoxicity	[103]
	Cytotoxicity	[189]
	Thioredoxin reductase inhibition	[71]
	Gastroprotective	[193]
	Cytotoxicity	[136]
	Cytotoxicity	[194]
	Anti-inflammatory	[194]
	Antiproliferative	[192]
	Cytotoxicity	[103]
**Jatropholone B**	Molluscicidal	[185]
	Gastroprotective	[99]
	Antiplasmodial	[90]
	Antituberculosis	[90]
	Cytotoxicity	[90]
	Cytotoxicity	[103]
	Thioredoxin reductase inhibition	[71]
	Gastroprotective	[193]
	Cytotoxicity	[136]
	Antiproliferative	[192]
	Cytotoxicity	[194]
	Anti-inflammatory	[194]
	Cytotoxicity	[103]
**Jatropholone C**	Cytotoxicity	[103]
**Jatropholone D**	Cytotoxicity	[103]
**Jatropholone E**	Cytotoxicity	[103]
**2α-hydroxyjatropholone**	Antiplasmodial	[90]
	Antituberculosis	[90]
	Cytotoxicity	[90]
**2β-hydroxyjatropholone**	Antiplasmodial	[90]
	Antituberculosis	[89]
	Cytotoxicity	[89]
**epi-jatrophol**	Cytotoxicity	[103]
	Cytotoxicity	[102]
**Jatrophaldehyde**	Cytotoxicity	[103]
	Cytotoxicity	[102]
**epi-jatrophaldehyde**	Cytotoxicity	[103]
	Cytotoxicity	[102]
	Antibacterial	[102]
**Jatromulone A**	Thioredoxin reductase inhibition	[71]
**Spruceanol**	Cytotoxicity	[195]

	Growth inhibition	[196]
	Cytotoxicity	[197]
	Cytotoxicity	[198]
	Antioxidant	[199]
	Cytotoxicity	[200]
	NO inhibition	[201]
	Cytotoxicity	[201]
	RANKL-induced osteoclastogenesis	[202]
	Cytotoxicity	[200]
	Antimicrobial	[203]
	Anti-HCV	[204]
	Cytotoxicity	[205]
	NO production inhibition	[206]
	Antitumoral	[207]
**Cleistanthol**	Cytotoxicity	[208]
	Antitumoral	[206]
	Cytotoxicity	[200]
	Antioxidant	[199]
	RANKL-induced osteoclastogenesis	[194]
	Cytotoxicity	[205]
	Antibacterial	[209]
	Antiviral	[209]
**Jatrocurcadiones A and B**	Thioredoxin reductase inhibition	[107]
**Lathyranlactone**	Cytotoxicity	[75]
**Jatrophodione A**	Cytotoxicity	[106]
	Cytotoxicity	[178]
	Thioredoxin reductase inhibition	[63]
**Jatrophodione B**	Cytotoxicity	[75]
**Jatrophodione C**	Cytotoxicity	[75]
**Jatrophodione D**	Cytotoxicity	[75]
**Jatrophodione E**	Cytotoxicity	[75]
**Jatrophalactam**	Cytotoxicity	[80]
**Heudelotinone**	Cytotoxicity	[106]
	Thioredoxin reductase inhibition	[70]
**Riolozatrione**	Cytotoxicity	[105]
	Antiviral (Antiherpes)	[105]
	Cytotoxicity	[210]
	Antiviral (Antiherpes)	[210]
**6-epi-riolozatrione**	Cytotoxicity	[105]
	Antiviral (Antiherpes)	[105]
**Jatrophatrione**	Antitumoral (Lymphocytic Leukemia)	[211]
	Cytotoxicity	[105]
	Antiherpes	[105]
**Citlalitrione**	Cytotoxicity	[105]
	Antiherpes	[105]
	Cytotoxicity	[100]
	Cytotoxicity	[212]
**Falodone**	Cytotoxicity	[213]
**Jatromultone A**	Cytotoxicity	[77]
**Jatromultone B**	Cytotoxicity	[77]
**Jatromultone C**	Cytotoxicity	[77]

**Jatromultone D**	Cytotoxicity	[77]
**Jatromultone F**	Cytotoxicity	[77]
**Jatromultone G**	Cytotoxicity	[77]
**Jatromultone H**	Cytotoxicity	[77]
**Jatromultone I**	Cytotoxicity	[77]
**Jatrogricaine A**	Anti-inflammatory	[77]
**Jatrofoliane A**	Reversal of multidrug resistance (MDR) in cancer cells	[81]
**Jatrofoliane B**	Reversal of multidrug resistance (MDR) in cancer cells	[81]
**Jatrophainolide A**	PTP1B enzyme inhibition (Type 2 diabetes and obesity)	[96]
**Jatrophainolide B**	PTP1B enzyme inhibition (Type 2 diabetes and obesity)	[96]
**Jatrophainolide C**	PTP1B enzyme inhibition (Type 2 diabetes and obesity)	[96]
**Jatrophacine**	Anti-inflammatory	[94]
**Integerrimene**	Cytotoxicity	[136]
**Jatrophadiketone**	Cytotoxicity	[78]
	Thioredoxin reductase inhibition	[70]
**Jaherin**	Antimicrobial and Antifungal	[214]
**Jatrophol**	Cytotoxicity	[103]
	Antibacterial	[187]
**Abiodone**	Cytotoxicity	[213]
**Curcusecon A**	Cytotoxicity	[88]
**Curcusecon B**	Cytotoxicity	[88]
**Curcusecon C**	Cytotoxicity	[88]
**Curcusecon D**	Cytotoxicity	[88]
**Curcusecon E**	Cytotoxicity	[88]

ADMET and Druglikeness

Expansions of figure 5. In the score plot, each compound makes up a sample, the codes are represented by three letters followed by numbers, the letters correspond to the name of each diterpene backbone and the number indicates the position that each one of them occupies in the dataset used to generate the molecular descriptors. This representation allows us to identify exactly which compound has different characteristics by interpreting the Score and Weight plots of the PCA.

Quadrant 1 –

- **A. Score plot**

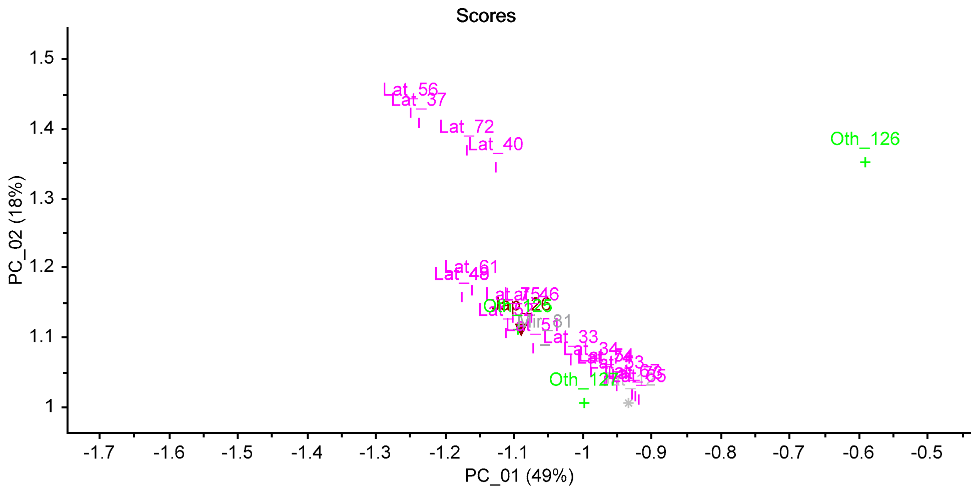

- **B. Weight plot**

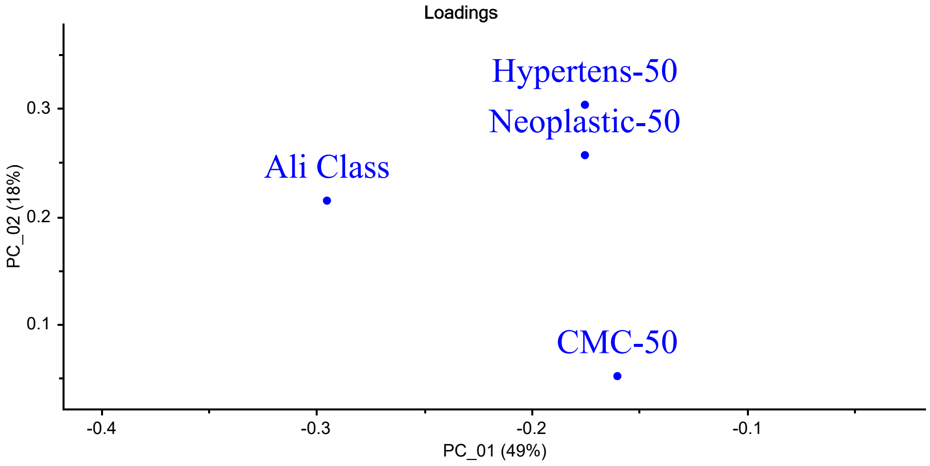

The expansion of the score plot shows the presence of diterpenes of the latirane type "Lat", in pink, concentrated in quadrant 1. In addition to these, in green there are a few compounds represented by the acronym "Oth", attributed to diterpenes of the seco or very modified series. It is also possible to observe substances of the myrsinane (Myr), jatrophane (Jat) and jatropholane (Jap) types. overlapping, which indicates their similarity in terms of physicochemical characteristics.

Quadrant 3 –

- **A. Score plot**

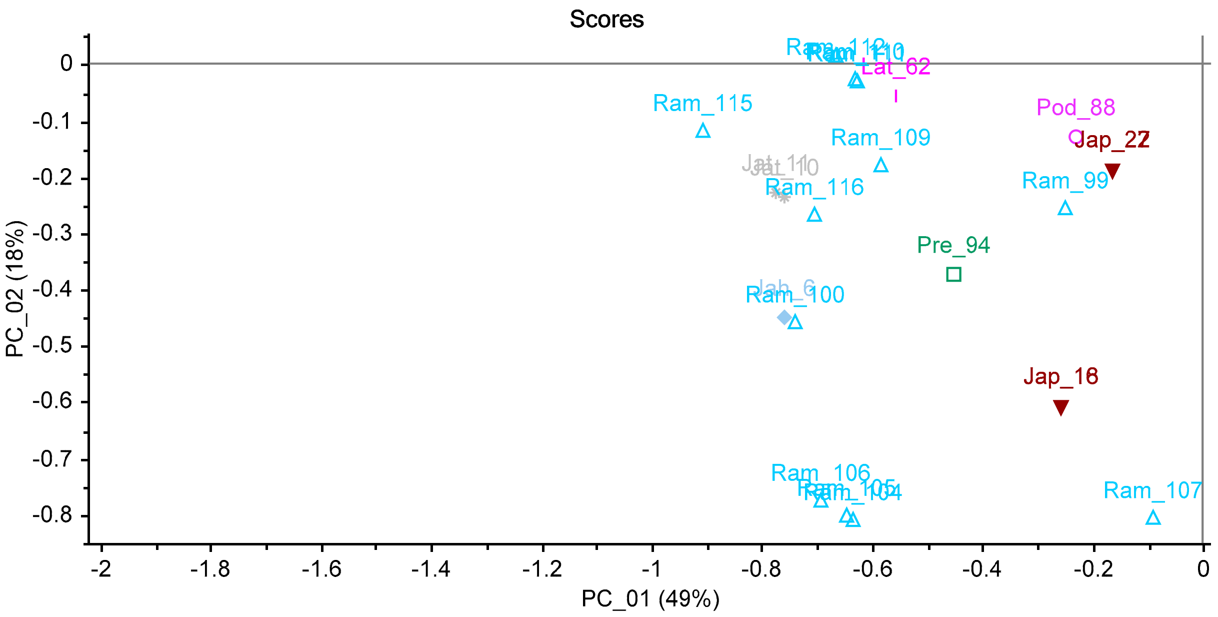

- **B. Weight plot**

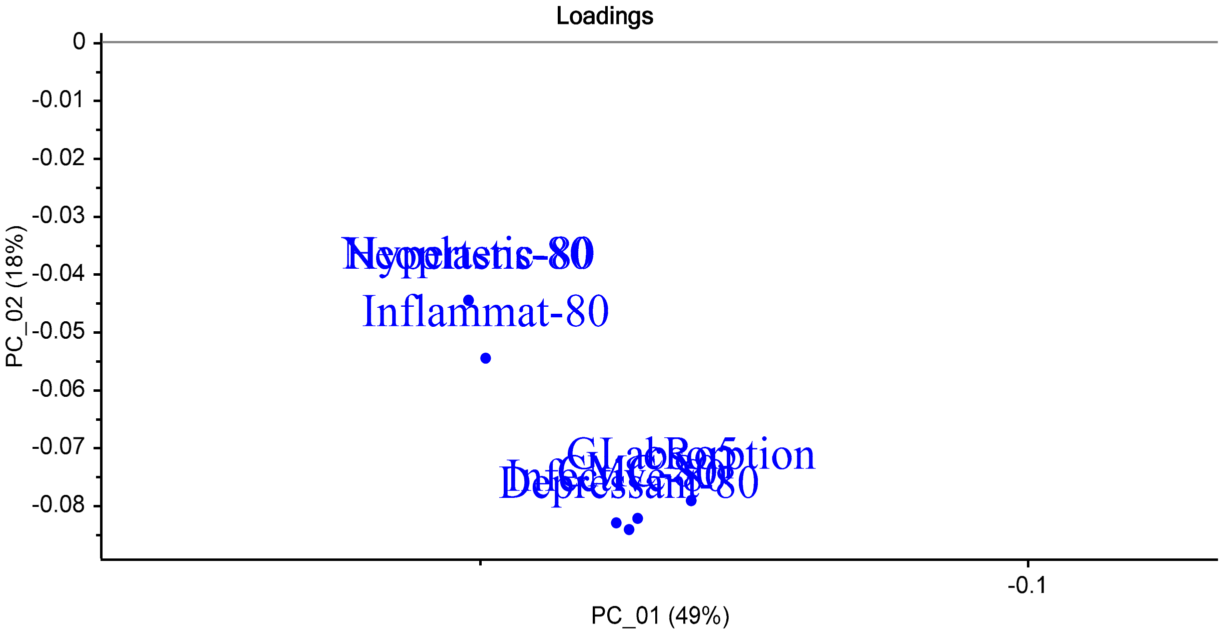

Quadrant 2 –

- **A. Score plot**

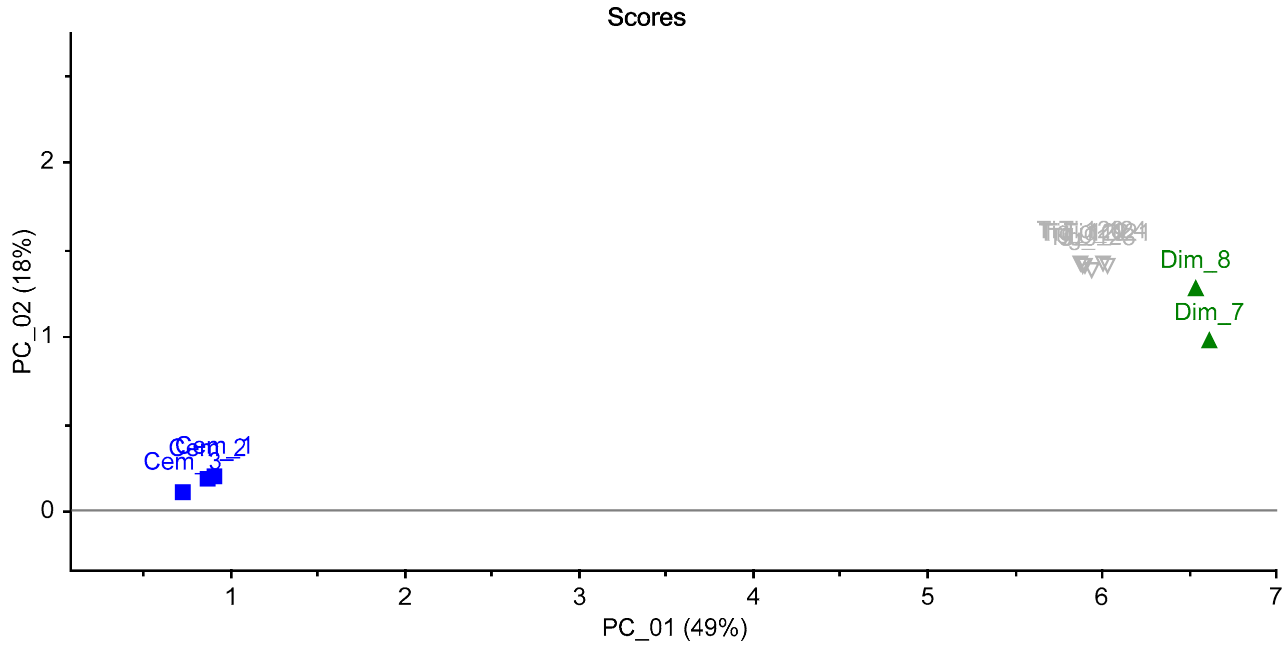

- **B. Weight plot**

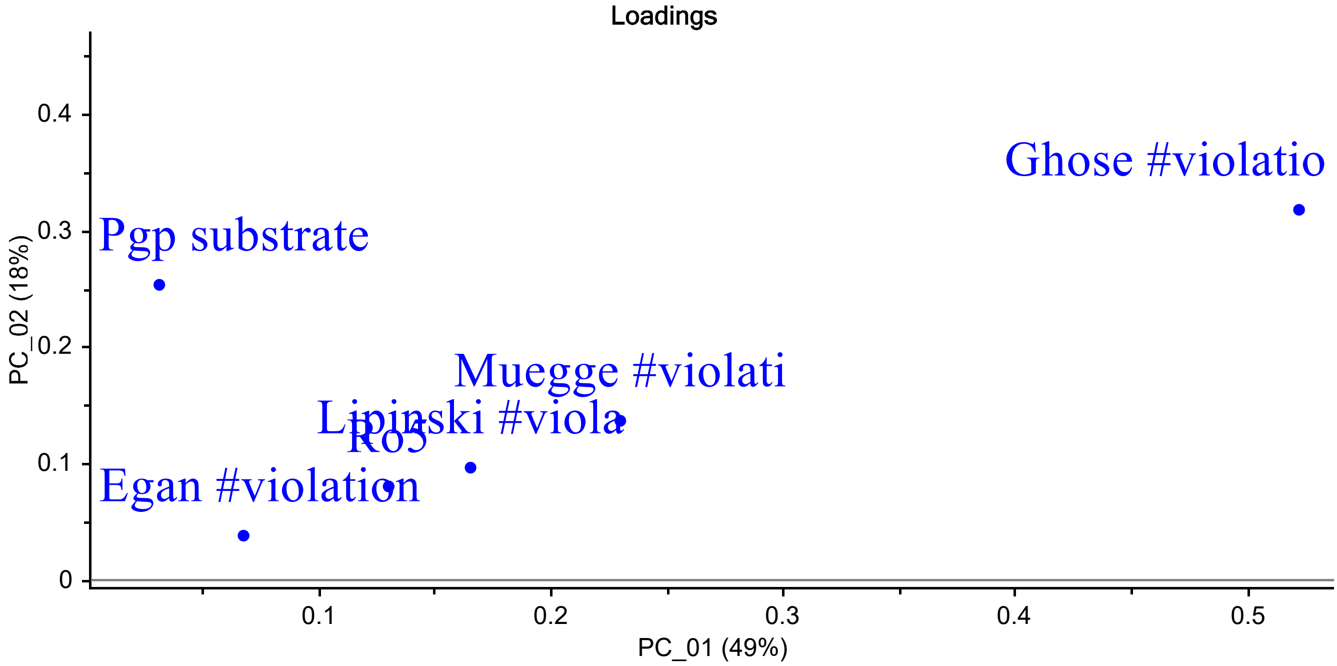

Quadrant 4

- **A. Score plot**

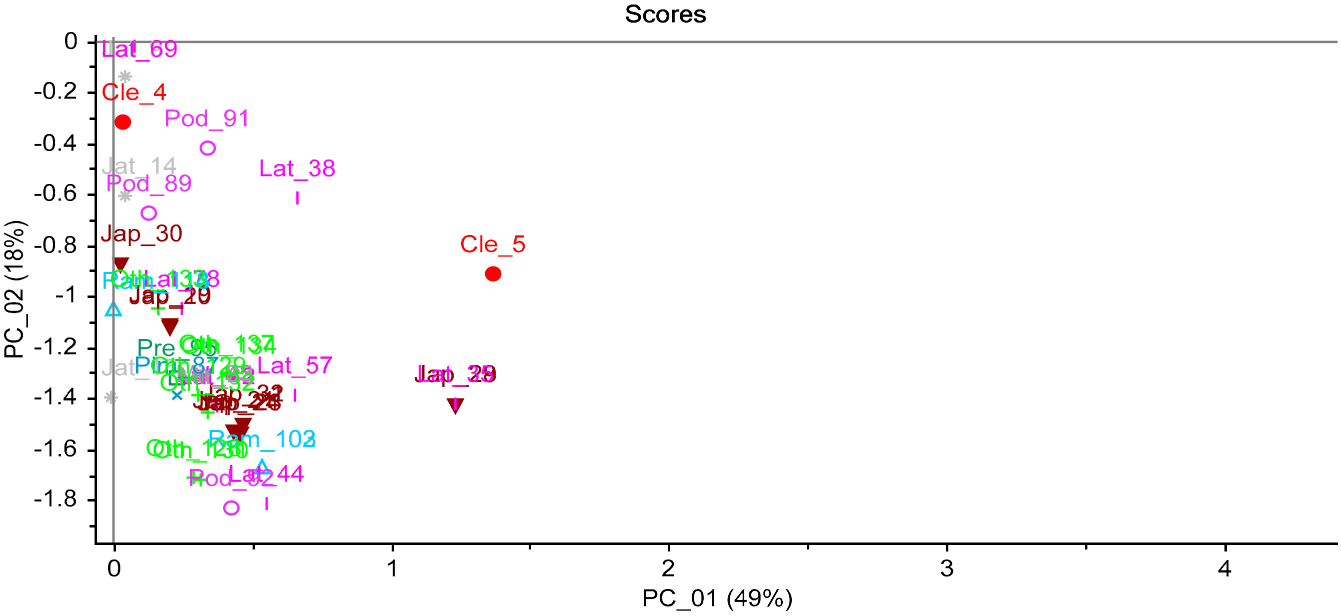

- **B. Weight plot**

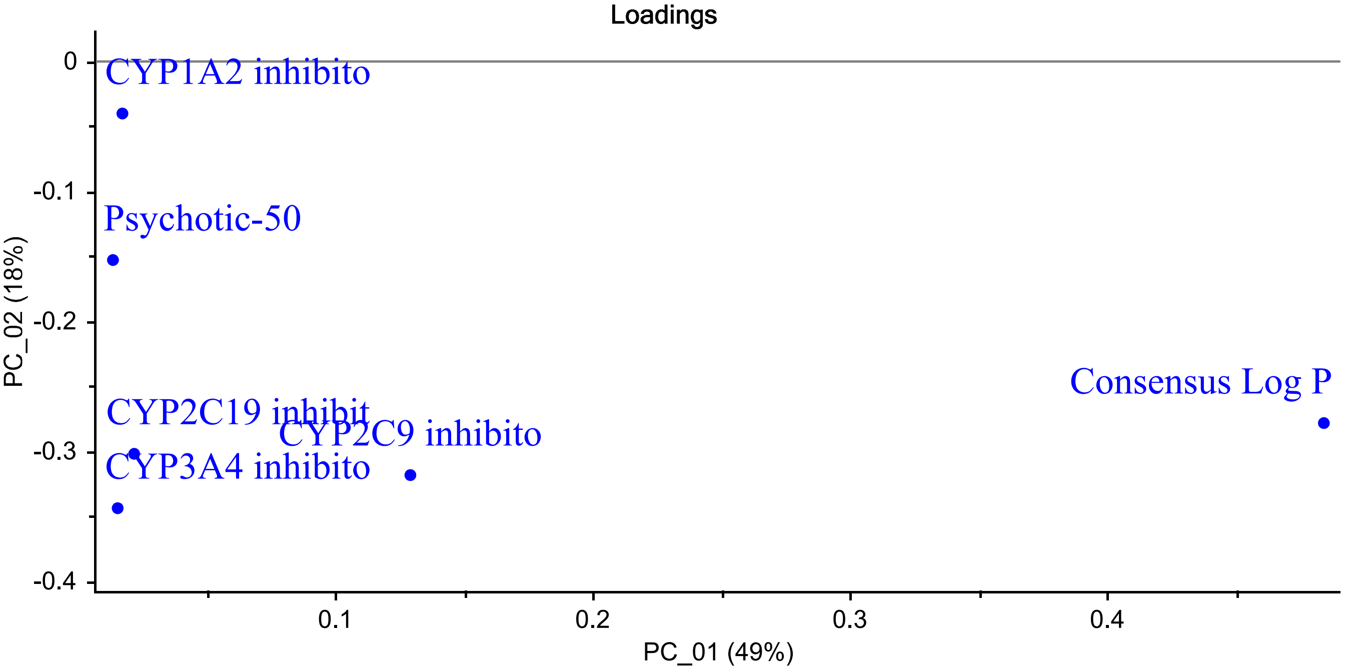

Supplement: Supplementary file 1 [file pharmaceuticals-17-01399-s001.zip › pharmaceuticals-3243211-supplementary.pdf]
